# Keratinocyte-derived cytokine TSLP promotes growth and metastasis of melanoma by regulating the tumor-associated immune microenvironment

**DOI:** 10.1172/jci.insight.161438

**Published:** 2022-11-08

**Authors:** Wenjin Yao, Beatriz German, Dounia Chraa, Antoine Braud, Cecile Hugel, Pierre Meyer, Guillaume Davidson, Patrick Laurette, Gabrielle Mengus, Eric Flatter, Pierre Marschall, Justine Segaud, Marine Guivarch, Pierre Hener, Marie-Christine Birling, Dan Lipsker, Irwin Davidson, Mei Li

**Affiliations:** 1 Institut de Génétique et de Biologie Moléculaire et Cellulaire (IGBMC), CNRS UMR 7104, Inserm U 1258, University of Strasbourg, Illkirch, France.; 2 Dermatology Clinic, Strasbourg University Hospital, Strasbourg, France.; 3 Institut Clinique de la Souris (ICS), Illkirch, France.

**Keywords:** Dermatology, Oncology, Cancer immunotherapy, Cellular immune response, Cytokines

## Abstract

Malignant melanoma is a major public health issue displaying frequent resistance to targeted therapy and immunotherapy. A major challenge lies in better understanding how melanoma cells evade immune elimination and how tumor growth and metastasis is facilitated by the tumor microenvironment. Here, we show that expression of the cytokine thymic stromal lymphopoietin (TSLP) by epidermal keratinocytes is induced by cutaneous melanoma in both mice and humans. Using genetically engineered models of melanoma and tumor cell grafting combined with TSLP-KO or overexpression, we defined a crosstalk between melanoma cells, keratinocytes, and immune cells in establishing a tumor-promoting microenvironment. Keratinocyte-derived TSLP is induced by signals derived from melanoma cells and subsequently acts via immune cells to promote melanoma progression and metastasis. Furthermore, we show that TSLP signals through TSLP receptor–expressing (TSLPR-expressing) DCs to play an unrecognized role in promoting GATA3^+^ Tregs expressing a gene signature including ST2, CCR8, ICOS, PD-1, CTLA-4, and OX40 and exhibiting a potent suppressive activity on CD8^+^ T cell proliferation and IFN-γ production. An analogous population of GATA3-expressing Tregs was also identified in human melanoma tumors. Our study provides insights into the role of TSLP in programming a protumoral immune microenvironment in cutaneous melanoma.

## Graphical abstract

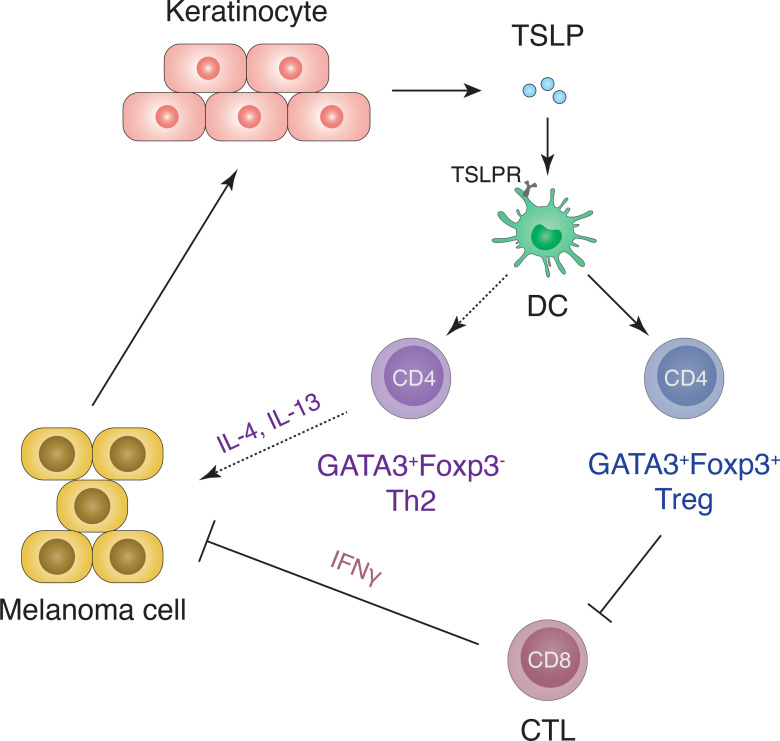

## Introduction

Cutaneous malignant melanoma is the most aggressive of human skin cancers, causing the majority (75%) of skin cancer–related deaths with an incidence of 15–25 per 100,000 (1). It remains a major public health issue due to increasing incidence, insufficient prognostic markers, and resistance to the developed MAP kinase inhibitor and immune checkpoint therapies. Immune cells in the tumor microenvironment (TME) not only fail to display effective antitumor properties, but also interact with tumor cells to aid in tumor growth and invasion (2). Tumors evade immune surveillance by multiple mechanisms. For example, one key mechanism is mediated through Tregs that critically contribute to the suppression of antitumor effects by immune cells and the generation of a protumorigenic TME. A high infiltration by Tregs versus non-Tregs has been associated with a poor prognosis and shorter overall survival for various types of human cancers, including melanoma (3). Immune checkpoint blockade that aims at restoring antitumor effects of immune cells has revolutionized the treatment of patients with advanced melanoma (4), but the number of responders is still limited. There is, thus, an urgent need to better understand how melanoma cells evade immune elimination and how tumor growth and metastasis is facilitated by the TME; it is also important to identify novel targets and predictive biomarkers for prognosis and clinical response.

Keratinocytes, the major cellular component in skin, actively participate in regulating skin and systemic immune responses, but their role in shaping the melanoma TME has been largely ignored. Thymic stromal lymphopoietin (TSLP), a pro-Th2 cytokine predominantly expressed by epithelial cells and keratinocytes, was initially recognized in driving pathogenesis of atopic diseases, including atopic dermatitis, asthma, and allergic march (5, 6), and it has been recently implicated in various types of tumors (7). TSLP-dependent Th2 type inflammation and tumor-promoting functions were reported in pancreatic (8) and breast cancers (9, 10), followed by a number of studies reporting protumor activity of TSLP in various tumors (reviewed in ref. 11). In contrast, conflicting data in breast cancer (12) and anti-tumor activity of TSLP in skin cancers (13, 14) were also reported. To our knowledge, no study has yet addressed the expression and role of TSLP in cutaneous melanoma.

We investigated the implication of TSLP in cutaneous melanoma growth and progression, using mouse melanoma models in which tamoxifen (Tam) treatment induces oncogenic Braf^V600E^ expression with or without ablation of the tumor suppressor Pten gene in melanocytes (15, 16), named hereafter Braf/Pten and Braf mice, respectively. Braf^V600E^, the most frequent activating mutation in melanoma found in 50%–60% of melanoma patients (17), activates the MAP kinase pathway and melanocyte proliferation, but it appears insufficient for generation of malignant melanoma. The combination of Braf^V600E^ together with silencing of Pten occurs in ~20% of melanoma patients (18) and, in the mouse model, leads to the development of primary melanoma with 100% penetrance, short latency, and metastasis to lymph nodes (LNs) (15). Using these melanoma models, we found that TSLP expression was induced in the epidermis concomitantly with tumorigenesis. By combining genetic TSLP ablation (19) with its drug-induced expression (19–21) in Braf/Pten or Braf mice, we showed that TSLP promotes melanoma progression and mediates an important crosstalk between melanoma cells, keratinocytes, and immune cells in establishing the melanoma TME. In addition, we identified a potentially novel role for TSLP, via TSLP receptor (TSLPR) expressed by DCs, in promoting a subset of GATA3-expressing Tregs, and we uncovered a potentially new facet to how TSLP regulates melanoma progression through programming TME. Moreover, analyses of melanoma patient biopsies and public human melanoma single-cell gene expression data provided further validation of mouse data in humans.

## Results

### Melanoma in Braf/Pten mice is associated with induction of TSLP in epidermis.

**Figure 1:**
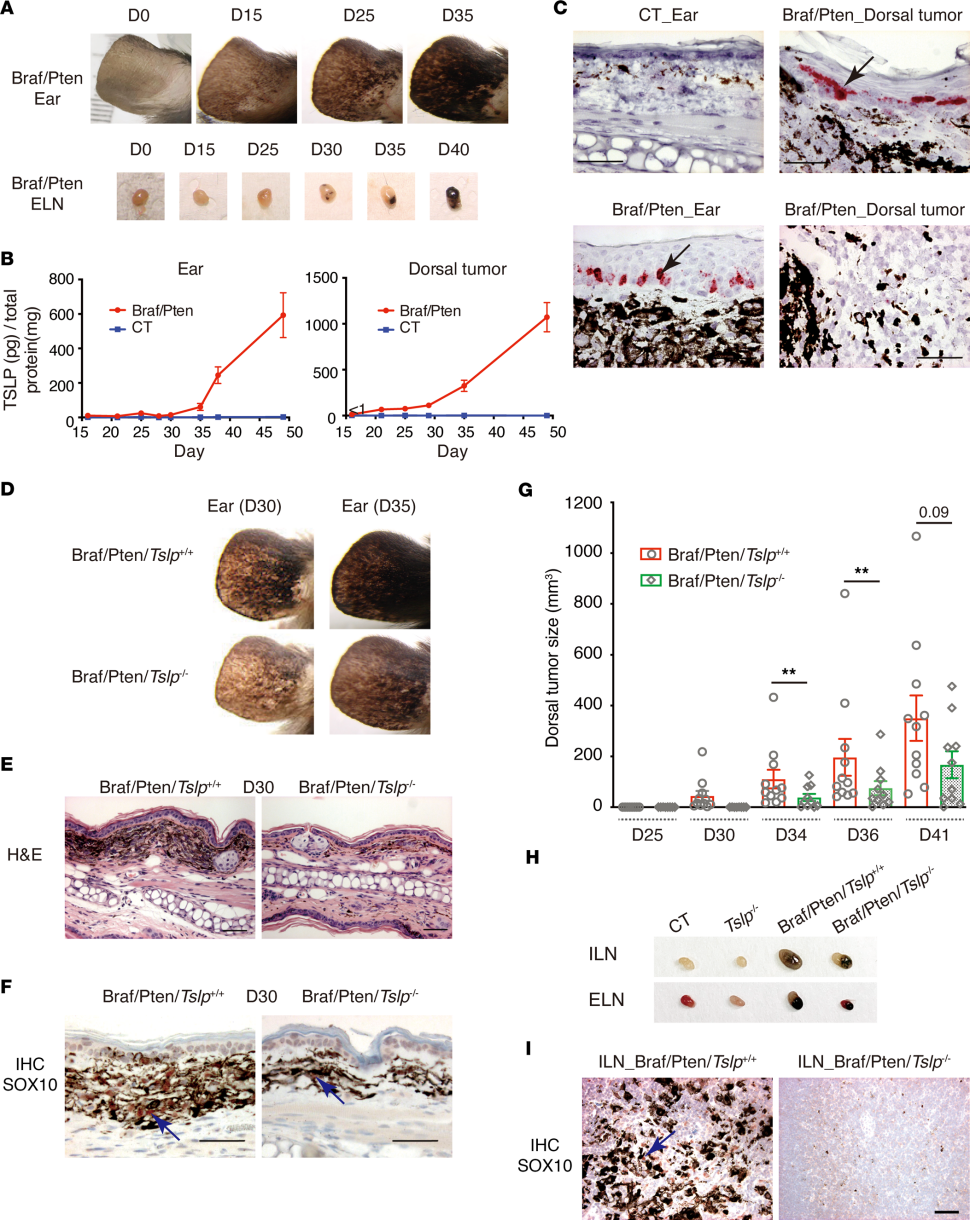
Keratinocyte-derived TSLP promotes growth and metastasis of Braf/Pten melanoma. (**A**–**C**) Epidermal TSLP expression is induced during melanomagenesis in Braf/Pten mice. (**A**) Ear and ear-draining lymph nodes (ELN) appearance of *Tyr:Cre^ERT2(tg/0)^ Braf^LSL–V600E/+^ Pten^lox/lox^* mice injected i.p. with tamoxifen (Braf/Pten mice). (**B**) Kinetic of TSLP protein levels in ears (left panel) and dorsal melanoma (right panel) from Braf/Pten mice, compared with ears and dorsal skin from control (CT, Tamoxifen-injected *Tyr:Cre^ERT2(0/0)^ Braf^LSL–V600E/+^ Pten^lox/lox^*) littermates. Data are shown as mean ± SEM. *n* = 5 for all groups, except *n* = 4 for Braf/Pten dorsal tumors and CT dorsal skin at D49. (**C**) RNAScope ISH for TSLP mRNA on paraffin sections. Black arrows point to one of the positive signals in the epidermis (in red). Scale bar: 50 μm. (**D**–**I**) Ablation of TSLP delays melanoma growth and metastasis. (**D**) Ear appearance of Braf/Pten/*Tslp*^+/+^ and Braf/Pten/*Tslp*^–/–^ mice at D30 and D35 following topical 4-hydroxytamoxifen (4-HT) treatment. (**E** and **F**) H&E staining (**E**) and IHC staining of SOX10 (**F**) of sections from ears at D30. Blue arrows points to one of the positive signals (in dark red). Scale bar: 50 μm. (**G**) Comparison of volumes of solid tumors developed in mice at the indicated time points. Data are shown as mean ± SEM. Student’s *t* test. **P < 0.01. *n* = 11 (Braf/Pten/*Tslp*^+/+^) and *n* = 10 (Braf/Pten/*Tslp*^–/–^). (**H**) Appearance of ELNs and dorsal tumor-draining inguinal lymph nodes (ILNs) at D41. (**I**) IHC staining of Sox10 on ILN sections at D41. Blue arrow points to one of the positive signals (in dark red). Scale bar: 50 μm. Data are representative of 3 independent experiments with similar results.

To examine the role of TSLP in melanoma, we generated *Tyr*:Cre-ER^T2(Tg/0)^ *Braf*^LSL–V600E/+^ *Pten*^lox/lox^ mice by breeding *Tyr*:Cre-ER^T2(tg/0)^ *Braf*^LSL–V600E/+^ (16) with *Pten*^lox/lox^ mice (22). Tam administration induced the expression of Braf^V600E^ and, at the same time, inactivated Pten expression selectively in melanocytes. Pigmented nevi started to appear at various body sites of Braf/Pten mice, including ears and dorsal and ventral skin from 2 weeks after Tam injection. On ears, pigmented cells started to appear at D15 and progressed to heavily pigmented lesions at D35 (Figure 1A) that were associated with pigmented metastases in draining LNs that accumulated with time (Figure 1A). On dorsal skin, pigmented nevi developed at D25, progressing to solid melanoma tumors at D35 (Supplemental Figure 1; supplemental material available online with this article; https://doi.org/10.1172/jci.insight.161438DS1), in agreement with previous reports (15, 23).

Ear and dorsal melanoma lesions from Braf/Pten mice were taken at different times, and TSLP protein levels were measured by ELISA. At both sites, TSLP was below detectable levels at D15 but increased from D20 (Figure 1B). In contrast, TSLP was not detected at any time in ears or dorsal skin from control mice (CT; Tam-treated *Tyr*:Cre^ERT2(0/0)^ *Braf*^LSL–V600E/+^ *Pten*^lox/lox^ mice) (Figure 1B). RNA in situ hybridization (ISH) showed that TSLP RNA was barely detected in ears or dorsal skin from CT mice (Figure 1C, upper left; not shown) but was strongly induced in the epidermis of ears and of dorsal tumors from Braf/Pten mice at D35 (Figure 1C, lower left and upper right). No or trace signals were observed inside solid tumors (Figure 1C, lower right). Reverse transcription quantitative real-time PCR (RT-qPCR) analyses of ear epidermis and dermis from Braf/Pten mice confirmed that TSLP was predominantly induced in the epidermal compartment (Supplemental Figure 2). These results indicate that TSLP expression was induced in the epidermal keratinocytes overlaying Braf/Pten melanomas during tumorigenesis.

### Genetic ablation of TSLP delays melanoma growth and metastasis.

To address the role of TSLP in melanoma, we generated Braf/Pten mice in the *Tslp^–/–^* background (named Braf/Pten/*Tslp*^–/–^) by breeding the *Tyr*:Cre-ER^T2^ *Braf*^LSL–V600E/+^ *Pten*^lox/lox^ mice with *Tslp^–/–^* mice (19). These mice were subjected to topical treatment with 4-hydroxytamoxifen (4-HT) to induce local melanoma lesions on ears or dorsal skin to avoid the incidence of melanoma all over the body, seen upon Tam injection.

Formation of pigmented lesions in the ears slowed and was less intense in Braf/Pten/*Tslp*^–/–^ mice, compared with Braf/Pten/*Tslp*^+/+^ mice (Figure 1D). H&E staining of ear sections at D30 showed there were less pigmented lesions in Braf/Pten/*Tslp*^–/–^ mice (Figure 1E). IHC staining of SOX10, a melanoma transcription factor expressed through early to late stages in this model (23, 24), confirmed that the pigmented cells corresponded to SOX10^+^ melanoma cells whose number was reduced in ears of Braf/Pten/*Tslp*^–/–^ mice (Figure 1F; see also Supplemental Figure 3 for cell counting). In addition, we evaluated the block of TSLP activity in Braf/Pten mice by administration of an anti-TSLP neutralization antibody (Supplemental Figure 4). While TSLP blockade led to a delayed appearance of pigmented lesion on ears, the effects were relatively weaker than seen using genetic ablation (Supplemental Figure 4). Subsequent studies were, thus, performed with mice with genetic ablation of TSLP.

On dorsal skin of Braf/Pten/*Tslp*^+/+^ mice, the local 4-HT treatment induced pigmented nevi appearing after 2 weeks that gradually progressed to pigmented and hypopigmented melanomas growing continuously with time (Supplemental Figure 5). The appearance of melanomas on dorsal skin was delayed, and their growth was diminished in Braf/Pten/*Tslp*^–/–^, as shown by comparison of their volumes at different times from D25 to D41 (Figure 1G). In addition, dorsal melanoma–draining inguinal LNs (ILNs) as well as ear-draining LNs (ELN) from Braf/Pten/*Tslp*^–/–^ mice were smaller and less pigmented compared with those from Braf/Pten/*Tslp*^+/+^ mice (Figure 1H). IHC staining of SOX10 showed a strong reduction in the number of SOX10^+^ metastatic cells in ILNs in Braf/Pten/*Tslp*^–/–^ mice (Figure 1I; see also Supplemental Figure 3 for cell counting). Taken together, these data indicate that genetic TSLP ablation slowed the growth and metastasis of melanoma in Braf/Pten mice.

### TSLP promotes growth of oncogenic Braf-driven melanoma and accelerates their metastasis.

**Figure 2:**
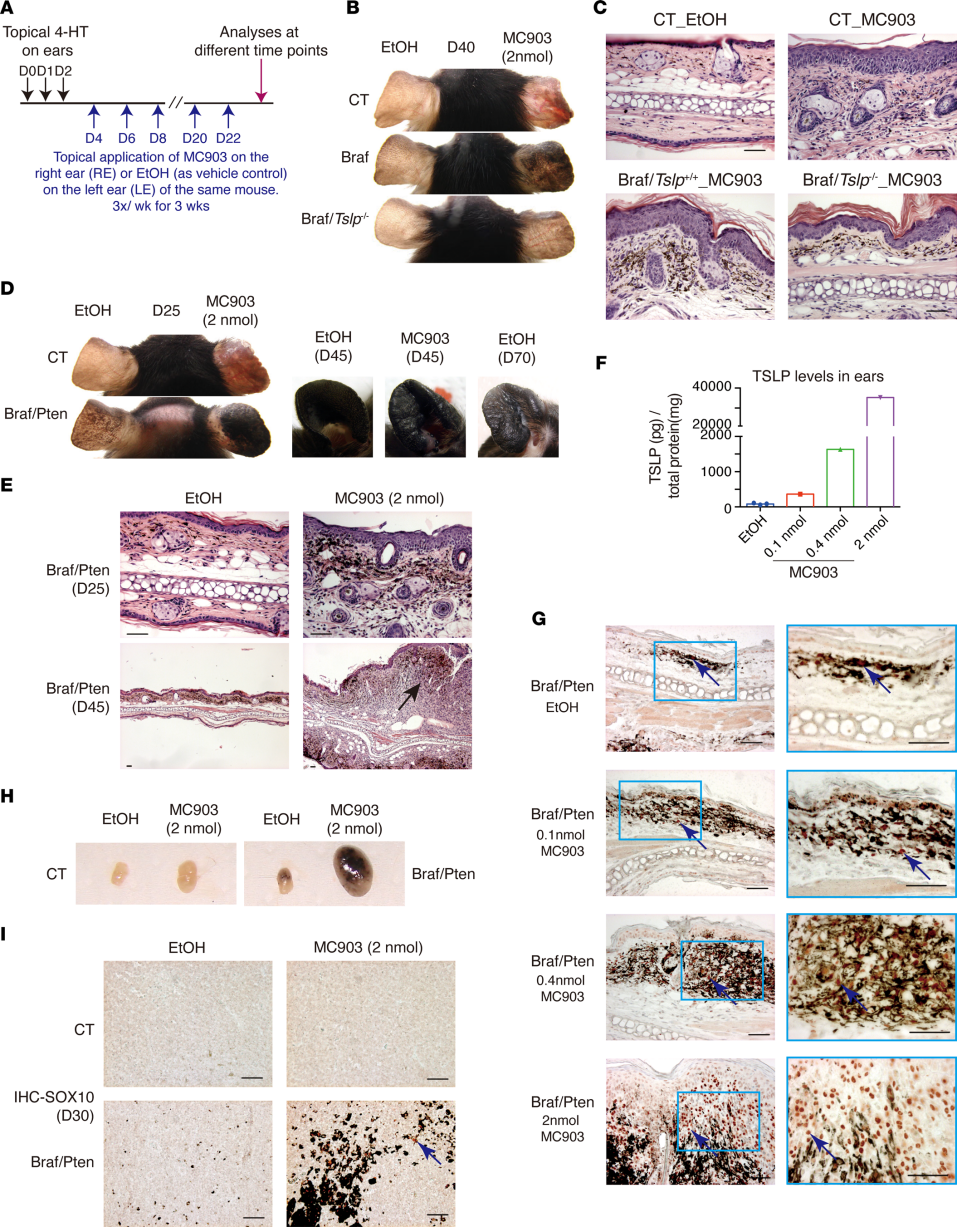
MC903-induced TSLP overexpression promotes and accelerates the growth and metastasis of mouse melanoma. (**A**) Experimental protocol. Dorsal part of ears of mice were topically treated with 4-hydroxytamoxifen (4-HT) for 3 days (D0–D2). Starting from D4, right ears (RE) were treated with MC903 (2 nmol), while left ears (LE) were treated with ethanol (EtOH, vehicle control) 3 times per week (wk) for 3 wks. Ears were analyzed at different time points. (**B**) Appearance of EtOH-treated LE and MC903-treated RE from CT, Braf, and Braf/*Tslp*^–/–^ mice at D40. (**C**) H&E staining of ear sections from EtOH-treated CT, MC903-treated CT, MC903-treated Braf, and MC903-treated Braf/*Tslp*^–/–^ mice. (**D**) Appearance of EtOH- and MC903-treated ears of CT and Braf/Pten mice at the indicated time points. (**E**) H&E staining of ear sections from EtOH- or MC903-treated Braf/Pten mice at D25 and D45. Note that the magnifications for photos of D25 and D45 are different, indicated by scale bars (50 μm for all). Black arrow points to the formation of solid tumor in MC903-treated Braf/Pten ears at D45. (**F**) TSLP protein levels in Braf/Pten mouse ears treated with EtOH or different doses of MC903 at D30. (**G**) IHC of SOX10 on ear sections of Braf/Pten mice treated with EtOH and different doses of MC903, at D30. Blue arrows point to one of the Sox10^+^ melanoma cells (in dark red). (**H** and **I**) Appearance of ear-draining lymph nodes (ELNs) (**H**) and IHC of Sox10 of ELNs (**I**) from EtOH- and MC903-treated CT and Braf/Pten mice at D30. Blue arrow points to one of the Sox10^+^ melanoma cells. Scale bar: 50 μm. Data are representative of 3 independent experiments with similar results.

In contrast to Braf/Pten mice, pigmented lesions developed very slowly on ears and dorsal skin of *Tyr*:Cre^ERT2(tg/0)^: *Braf*^LSL–V600E/+^ (Braf mice), appearing 2 months after Tam injection or topical 4-HT treatment with melanomas barely observed within 6 months, as previously reported (16). Correspondingly, TSLP was not detectable by ELISA in 4-HT–treated ears or dorsal skin of Braf mice at D40 or D60 (data not shown). To test the effect of TSLP expression on the growth of Braf melanoma, we employed our previously established experimental protocol where topical application of the low calcemic vitamin D analog MC903 on mouse skin potently induces TSLP expression in epidermal keratinocytes, triggering TSLP-dependent skin inflammation (19–21). Following topical 4-HT treatment, mice were treated with 2 nmol MC903 on right ears (RE) and with ethanol (EtOH, vehicle control) on left ears (LE) (Figure 2A). Strikingly, at D40, EtOH-treated LEs did not show any sign of pigmentation, whereas MC903-treated REs already developed pigmented lesions (Figure 2, B and C). Note that MC903 treatment on CT ears did not result in any pigmentation, although the ears became red and inflamed due to TSLP-induced skin inflammation as previously reported (19, 21). Importantly, the acceleration of pigmentated lesion formation and melanoma cell growth by MC903 was abolished in Braf/*Tslp*^–/–^ mice (Figure 2, B and C), indicating that these effects were mediated through TSLP.

We next tested whether MC903 treatment would further accelerate tumor growth and metastasis in Braf/Pten mice. Indeed, upon MC903 treatment, pigmented lesions appeared earlier and progressed faster in ears of Braf/Pten mice. At D25, the MC903-treated RE exhibited more advanced skin pigmentation compared with the EtOH-treated LE, which reached a similar appearance only at D45 (Figure 2D). In addition, the pigmented lesions in MC903-treated Braf/Pten ears quickly progressed to melanoma tumors at D45, whereas analogous melanomas were observed on EtOH-treated ears at a much later stage (at D70) (Figure 2D). We did not observe any hyperpigmentation on MC903-treated ears from CT mice at any time points examined (Figure 2D and data not shown). H&E staining showed that MC903-treated ears exhibited an increased number of pigmented cells in the dermis at D25 and also exhibited a formation of melanomas growing deep in the dermis at D45 (Figure 2E). Moreover, upon MC903 treatment, many more pigmented and nonpigmented (neural-crest stem cell like; ref. 23) Sox10^+^ melanoma cells were observed at D45 compared with EtOH-treated ears (Supplemental Figure 6). MC903 treatment, thus, accelerated progression of Braf/Pten melanoma.

We previously showed that MC903 induces TSLP expression in epidermal keratinocytes in a dose-dependent manner (25), providing an opportunity to test whether melanoma growth could be promoted dose dependently by TSLP. Treatment with MC903 at 0.1 nmol, 0.4 nmol, or 2 nmol on Braf/Pten ears induced increasing levels of TSLP (Figure 2F) that consequently promoted development of melanoma lesions shown by increasing numbers of SOX10^+^ pigmented and nonpigmented melanoma cells in the dermis (Figure 2G; see also Supplemental Figure 3 for cell counting). Braf/Pten melanoma cell growth was, thus, accelerated by TSLP in a dose-dependent manner.

We further examined whether melanoma metastasis to draining LNs was also promoted by MC903-induced TSLP expression. ELNs from EtOH- or MC903-treated CT mice did not exhibit any pigmentation (Figure 2H), whereas in Braf/Pten mice, ELNs draining MC903-treated ears were significantly bigger and hyperpigmented compared with EtOH-treated ears (Figure 2H). No SOX10^+^ cells were seen in ELNs from EtOH- or MC903-treatment CT mice (Figure 2I). In contrast, ELNs draining the MC903-treated Braf/Pten ears exhibited many SOX10^+^ melanoma cells (Figure 2I; see also Supplemental Figure 3 for cell counting), whereas they were much less abundant in ELNs draining the EtOH-treated ears. Topical application of MC903, therefore, accelerated local growth of melanoma cells and their metastasis to LNs.

Taken together, these results indicate that activation of TSLP expression in the epidermis promoted growth of Braf melanoma and accelerated the progression and metastasis of Braf/Pten melanoma.

### TSLP promotes growth of skin-grafted B16F10 melanoma cells through immune cells.

**Figure 3:**
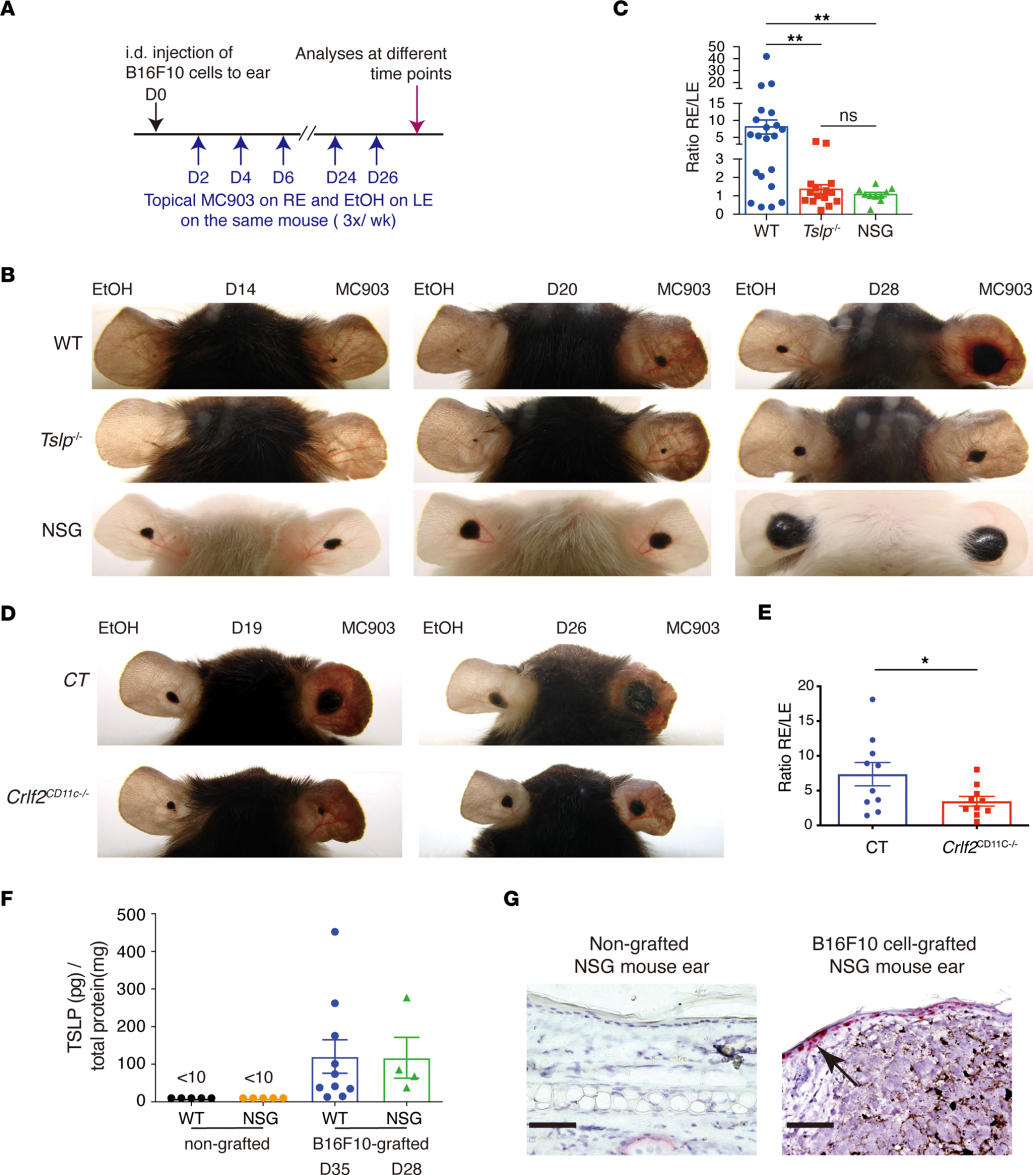
Crosstalk of B16F10 melanoma cells, keratinocytes, and immune cells. (**A**–**C**) TSLP promotes B16F10 melanoma cell growth through immune cells. (**A**) Treatment protocol. After B16F10 cell i.d. grafting, mice were treated with MC903 on right ears (RE) or with ethanol (ETOH) on left ears (LE) 3 times per week (wk) for 4 wks. (**B**) Appearance of B16F10-grafted ears of WT, *Tslp^–/–^*, and NSG mice. (**C**) Tumor areas on ears were measured by ImageJ, and the ratio of tumor area between RE and LE from the same mouse was calculated. Each dot represents the ratio from 1 individual mouse. Data are shown as mean ± SEM. *n* = 21 (WT); *n* = 15 (*Tslp*^–/–^); *n* = 11 (NSG). One-way ANOVA test. **P < 0.01. ns, nonsignificant. (**D** and **E**) MC903-promoted B16F10 melanoma is reduced in mice with selective ablation of TSLPR in DCs. (**D**) Appearance of EtOH-treated LEs and MC903-treated REs of CD11c-Cre^0/0^*Crlf2*^L2/L2^ (control, CT) and CD11c-Cre^Tg/0^*Crlf2*^L2/L2^ (*Crlf2*^CD11c–/–^) mice. (**E**) Ratio of tumor area between RE and LE. *n* = 10. Student’s *t* test. *P < 0.05. (**F** and **G**) B16F10 cell grafting induces TSLP expression in NSG mice. (**F**) B16F10 cells were i.d. injected into ears of WT or NSG mice at D0. TSLP protein levels measured by ELISA in B16F10-grafted ears of WT (at D35) and NSG (at D28 when melanoma reached similar size as WT in D35) mice, compared with nongrafted WT or NSG ears. (**G**) RNAScope ISH for TSLP mRNA in NSG and B16F10-grafted NSG ears at D28. Black arrows point to one of the positive signals (in red). Scale bar: 50 μm. *n* = 5 (nongrafted WT or NSG); *n* = 10 (B16F10-grafted WT); *n* = 4 (B16F10-grafted NSG). All data are representative of 3 independent experiments with similar results.

We next asked whether TSLP promoted melanoma growth by acting directly on melanoma cells or through immune cells. To examine this, we intradermally (i.d.) grafted B16F10 melanoma cells into ears of WT C57BL/6J mice or immunodeficient NOD.*Cg*-P*rkdc^scid^Il2rg*^tm1Wjl^/SzJ (NSG) mice that lack mature T, B, NK, and innate lymphoid cells (ILCs) (26). We treated REs of WT, *Tslp^–/–^*, and NSG mice with MC903 and LEs with EtOH following the grafting of B16F10 cells and monitored subsequent melanoma growth (Figure 3A). MC903 promoted growth of B16F10 melanoma cells grafted in WT ears, with tumor areas on MC903-treated REs significantly larger than those on EtOH-treated LEs (Figure 3B; compare LE and RE of WT mice). In contrast, MC903 did not promote B16F10 cell growth grafted to *Tslp^–/–^* mice (Figure 3B; compare LE and RE of *Tslp^–/–^* mice). Calculation of tumor cell areas on REs and LEs showed that the RE/LE ratio was much higher in WT mice compared with *Tslp^–/–^* mice (Figure 3C), demonstrating that MC903 promoted B16F10 cell growth through TSLP, in keeping with the above data from Braf and Braf/Pten mice. Moreover, B16F10 grafted in NSG mice grew faster than in WT mice, likely due to their immunodeficiency, but MC903 treatment failed to have any further promoting effect (Figure 3, B and C). Together, these results indicate that TSLP did not act directly on B16F10 cells to stimulate their growth; instead, TSLP-stimulated growth required the presence of an intact immune cell population absent in the NSG background.

### TSLP signals through TSLPR expressed by DCs to exert its promoting effect on B16F10 tumour growth.

TSLP signals through the TSLP receptor expressed by a variety of immune cells including DCs that have been implicated in TSLP-driven Th2 and T follicular helper (Tfh) cell responses in mice (20, 27) and humans (28, 29). To investigate whether the tumor-promoting effect of TSLP involves signaling through TSLPR expressed by DCs, we generated mice with the ablation of TSLPR selectively in DCs by breeding mice bearing the floxed allele of the *Crlf2* gene with CD11c-Cre transgenic mice (30). CD11c-Cre^Tg/0^/*Crlf2*^L2/L2^ mice (named as *Crlf2*^CD11c–/–^) together with their CD11c-Cre^0/0^/*Crlf2*^L2/L2^ CT littermates were i.d. grafted with B16F10 cells, followed by MC903 treatment on RE and EtOH on LE, as shown in Figure 3A. Comparison of tumor cell areas in RE and LE showed that the RE/LE ratio was reduced in *Crlf2*^CD11c–/–^ mice (Figure 3, D and E), suggesting that TSLPR expressed by DCs was crucially required for the tumor-promoting effect of TSLP.

### B16F10 cell grafting induces TSLP production in immunodeficient NSG mice.

On the other hand, we investigated whether TSLP was induced by signals derived from melanoma cells or from immune cells. ELISA measurement showed that TSLP protein levels were elevated in B16F10-grafted WT or NSG ears in presence of melanomas (Figure 3F; D35 for WT and D28 for NSG mice). RNAScope ISH showed that TSLP RNA was detected in the epidermis from B16F10 cell-grafted NSG mouse ears but was barely detected in nongrafted NSG mouse ears (Figure 3G). TSLP induction in epidermal keratinocytes by engrafted B16F10 melanomas did not, therefore, appear to require the immune cells that are absent in NSG mice. Moreover, TSLP expression was increased after floating culture of the epidermis from WT mice on the medium of plated B16F10 cells (Supplemental Figure 7), supporting the idea that epidermal TSLP expression was induced directly by signals derived from tumor cells.

Together, the above data suggest that melanoma-derived signals induced TSLP expression in keratinocytes that, in turn, signals through DCs expressing TSLPR to trigger immune cascades promoting melanoma growth and metastasis.

### Genetic ablation of TSLP reduces GATA3^+^ Tregs in LNs draining Braf/Pten melanoma.

**Figure 4:**
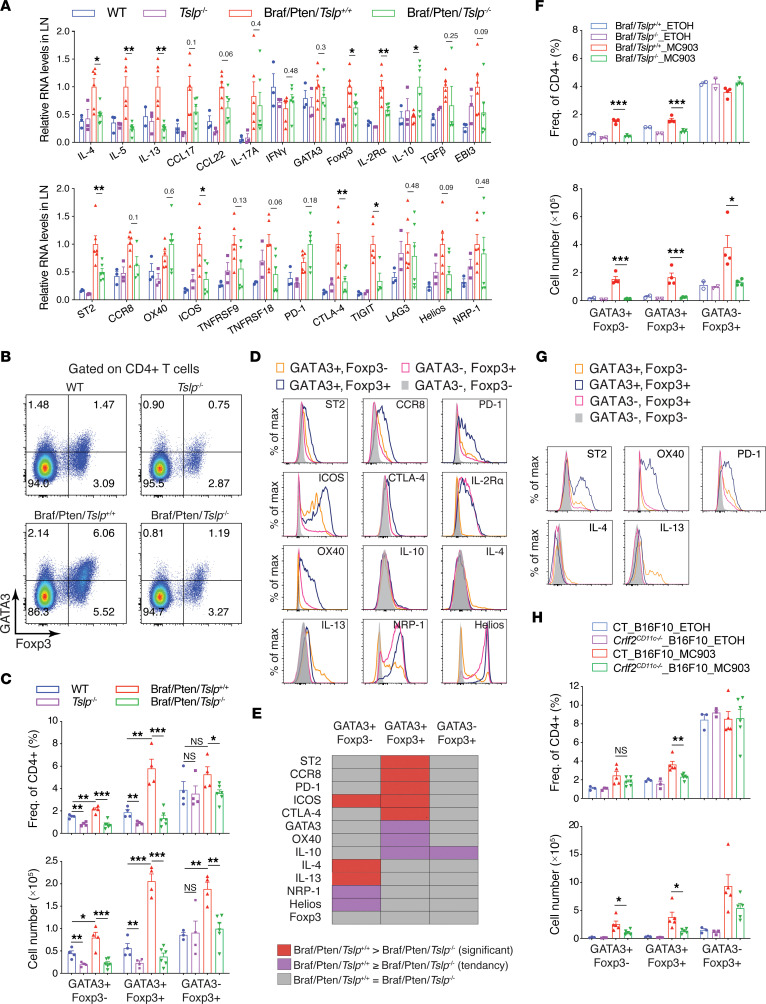
TSLP promotes GATA3^+^ Tregs in melanoma-draining lymph nodes. (**A**) qPCR analyses. Data are shown as mean ± SEM. Student’s *t* test. *n* = 3 for WT and *Tslp*^–/–^ groups; *n* = 4–6 for Braf/Pten/*Tslp*^+/+^ and Braf/Pten/*Tslp*^–/–^ groups. (**B** and **C**) Flow cytometry analyses of GATA3 and Foxp3 among CD4^+^ T cells, showing representative FACS plots (**B**), as well as their frequencies and cell numbers (**C**). One-way ANOVA test. *n* = 3–4 (WT and *Tslp*^–/–^); *n* = 4 (Braf/Pten/*Tslp*^+/+^); *n* = 6 (Braf/Pten/*Tslp*^–/–^). (**D**) Histogram presentation for indicated markers analyzed in the GATA3/Foxp3 CD4^+^ T cell populations. (**E**) A summary for the pattern of median fluorescence intensity (MFI) comparisons of the examined markers in the indicated cell populations between Braf/Pten/*Tslp*^+/+^ and Braf/Pten/*Tslp*^–/–^ LNs. Red, significantly higher in Braf/Pten/*Tslp*^+/+^ than Braf/Pten/*Tslp*^–/–^. Purple, tendency to be higher in Braf/Pten/*Tslp*^+/+^ than Braf/Pten/*Tslp*^–/–^. Gray, equal between Braf/Pten/*Tslp*^+/+^ and Braf/Pten/*Tslp*^–/–^. (**F**) Frequencies and cell numbers of GATA3/Foxp3 CD4^+^ T cells in ELNs of Braf/*Tslp*^+/+^ and Braf/*Tslp*^–/–^ treated with ETOH or MC903, as indicated in Figure 2A. Student’s *t* test. *n* = 2 (ETOH groups); *n* = 4 (MC903 groups). (**G**) Histogram presentation for indicated markers analyzed in the GATA3/Foxp3 CD4 T cell populations in ELNs of MC903-treated Braf/*Tslp*^+/+^ mice. (**H**) Frequencies and cell numbers of GATA3/Foxp3 CD4^+^ T cells in ELNs of CT (CD11c-Cre^0/0^*Crlf2*^L2/L2^) and *Crlf2*^CD11c–/–^ (CD11c-Cre^Tg/0^*Crlf2*^L2/L2^) mice grafted with B16F10 cells and treated with ETOH or MC903, as indicated in Figure 3A. Student’s *t* test. *n* = 3 (ETOH groups); *n* = 5 (MC903 groups). All data are representative of more than 3 independent experiments with similar results. *P < 0.05; **P < 0.01.

We and others previously demonstrated that epidermal TSLP is a key driver of the Th2 inflammatory response in mice (19, 21, 31, 32). We examined Th2 response–associated gene expression by qPCR in the ILNs draining dorsal skin of WT and *Tslp*^–/–^ mice and draining dorsal melanomas of Braf/Pten/*Tslp*^+/+^ and Braf/Pten/*Tslp*^–/–^ mice. Expression of Th2-type cytokines IL-4, IL-5, and IL-13 and chemokines CCL17 and CCL22 was upregulated in Braf/Pten/*Tslp*^+/+^ mice compared with WT mice, but it was diminished in Braf/Pten/*Tslp*^–/–^ mice, in agreement with the recognized role of TSLP in promoting Th2 response (Figure 4A). In contrast, RNA levels of Th17 type cytokine IL-17A were higher in Braf/Pten/*Tslp*^+/+^ than in WT mice but remained unchanged in Braf/Pten/*Tslp*^–/–^ (Figure 4A). The expression of the Th1-type cytokine IFN-γ did not exhibit clear change among the groups (Figure 4A). Nevertheless, expression of Treg-associated genes Foxp3 and IL2Rα (CD25) was increased in Braf/Pten/*Tslp*^+/+^ mice compared with CT mice; interestingly, this increase was strongly diminished in Braf/Pten/*Tslp*^–/–^ mice (Figure 4A). This observation prompted us to further analyze gene sets related to Tregs — particularly, tumor-associated Tregs recently identified in various tumor contexts, variously designated as intratumoral Tregs, activated Tregs, or effector Tregs (33–37) — that express specific signatures including cytokine/chemokine receptors ST2 (IL1RL1) and CCR8; immune checkpoints CTLA-4, PD-1 (PDCD1), ICOS, and OX40 (TNFRSF4); and cytokines IL-10, TGF-β, and IL-35. Intriguingly, among the genes examined, we observed that ST2, CCR8, ICOS, TNFRSF9 (4-1BB), TNFRSF18 (GITR), PD-1, CTLA-4, TIGIT, LAG3, Helios (IKZF2), NRP-1, TGF-β, and EBI3 (subunit of IL-35) all showed increased expression in Braf/Pten/*Tslp*^+/+^ compared with WT (Figure 4A). Of these genes, some (ST2, ICOS, CTLA-4, TIGIT) showed a strong reduction, and others (CCR8, TNFRSF9, TNFRSF18, TGF-β, EBI3) showed a milder reduction in Braf/Pten/*Tslp*^–/–^ compared with Braf/Pten/*Tslp*^+/+^ mice. Thus, these analyses suggest that, in addition to its long-recognized Th2 promoting role, TSLP may have a role in tumor-associated Treg response.

We next examined Th2 and Tregs in draining LNs by performing intracellular staining and flow cytometry analyses for GATA3 and Foxp3, the characteristic transcription factors for Th2 and Tregs, respectively. Gating with GATA3 and Foxp3 on CD4^+^ T cells distinguished 4 populations: GATA3**^+^**Foxp3**^–^**, GATA3**^+^**Foxp3^+^, GATA3**^–^**Foxp3**^+^**, and GATA3**^–^**Foxp3**^–^** (Figure 4B). We observed a significant increase in both frequencies and numbers of GATA3^+^Foxp3^–^ and GATA3^+^Foxp3^+^ cells in Braf/Pten*/Tslp*^+/+^ compared with WT mice and all were reduced in Braf/Pten/*Tslp*^–/–^ mice (Figure 4, B and C). Although the overall cell number appeared to be increased, the relative proportion of GATA3^–^Foxp3^+^ cells was not significantly increased in Braf/Pten/*Tslp*^+/+^ mice compared with CT mice (Figure 4, B and C), and their frequencies and numbers were lower in Braf/Pten/*Tslp*^–/–^ mice (Figure 4, B and C).

To further explore the nature of GATA3^+^Foxp3^–^, GATA3^+^Foxp3^+^, and GATA3^–^Foxp3^+^ cells, we examined additional markers for Treg and Th2 cells in ILNs draining Braf/Pten/*Tslp*^+/+^ dorsal melanomas. GATA3^+^Foxp3^+^ and GATA3^–^Foxp3^+^ both presented a high expression of IL-2R, Helios, and NRP-1, suggesting the Treg nature of these 2 populations, named as GATA3^+^ Tregs and GATA3^–^ Tregs, respectively (Figure 4D). Strikingly, GATA3^+^ Tregs presented the highest expression of ST2, CCR8, ICOS, PD-1, CTLA-4, and OX40 among the 4 populations, notably higher than in GATA3^–^ Tregs (Figure 4D), indicating that these markers represent the signatures of GATA3^+^ Tregs. Despite GATA3 expression, we did not detect Th2 cytokines IL-4 and IL-13 in GATA3^+^ Tregs (Figure 4D). Instead, these 2 cytokines were specifically detected in GATA3^+^Foxp3^–^ cells (Figure 4D), suggesting that these cells represented Th2 (named GATA3^+^ Th2). In addition, highest levels of IL-10 were observed in GATA3^+^ Tregs, followed by GATA3^–^ Tregs and GATA3^+^ Th2 cells, although levels remained weak for all populations (Figure 4D). In Braf/Pten melanoma-draining LNs, TSLP promoted not only GATA3^+^ Th2 cells, but also GATA3^+^ Tregs expressing a specific signature including ST2, CCR8, ICOS, PD-1, CTLA-4, OX40, and IL-10 — but not Th2 cytokines IL-4 and IL-13.

To investigate the role of TSLP, we compared the expression of these signature markers in GATA3^+^ Tregs, as well as GATA3^–^ Tregs and GATA3^+^ Th2 cell populations between Braf/Pten/*Tslp*^+/+^ and Braf/Pten/*Tslp*^–/–^ mice. In GATA3^+^ Tregs (Figure 4E and Supplemental Figure 8), expression of signature genes ST2, CCR8, PD-1, ICOS, and CTLA-4 were all significantly reduced in Braf/Pten/*Tslp*^–/–^ compared with Braf/Pten/*Tslp*^+/+^. Expression of GATA3, OX40, and IL-10 also tended to be lower in Braf/Pten/*Tslp*^–/–^, showing that their expression in GATA3^+^ Tregs is TSLP dependent. In GATA3^+^ Th2 cells, we observed that expression of IL-4 and IL-13, as well as ICOS, were also lower in Braf/Pten/*Tslp*^–/–^ compared with Braf/Pten/*Tslp*^+/+^, suggesting a TSLP dependence. Notably, none of the examined markers exhibited TSLP dependency in GATA3^–^ Tregs. Thus, these data indicate that, in Braf/Pten melanoma-draining LNs, TSLP regulated gene expression in GATA3^+^ Tregs and GATA3^+^ Th2 populations.

### Overexpression of TSLP drives GATA3^+^ Tregs through TSLPR-expressing DCs.

Since increased TSLP induced by topical MC903 treatment promoted pigmented lesions in Braf mouse ears (Figure 2, B and C), we examined whether MC903 treatment could drive GATA3^+^ Tregs in ELNs from Braf mice. Flow cytometry analyses of CD4^+^ T cells showed that MC903 treatment led to increased numbers of GATA3^+^ Tregs, as well as GATA3^+^ Th2 cells, in ELNs from Braf/*Tslp*^+/+^ mice, but not Braf/*Tslp*^–/–^ mice (Figure 4F). Increased TSLP, thus, promoted generation of GATA3^+^ Tregs and GATA3^+^ Th2 in MC903-treated Braf mice. Similar to what was observed in Braf/Pten tumor-draining ILNs, MC903 treatment–promoted GATA3^+^ Tregs in Braf ELNs exhibited signature expression of ST2, OX40, and PD-1 (Figure 4G), but not IL-4 and IL-13 which were only detected in GATA3^+^ Th2 cells (Figure 4G). These results, thus, indicate that epidermal TSLP overexpression promoted GATA3^+^ Tregs in addition to GATA3^+^ Th2 in draining LNs from Braf mice.

We further examined whether TSLP promotes GATA3^+^ Tregs and GATA3^+^ Th2 cells through TSLPR-expressing DCs. *Crlf2*^CD11c–/–^ and CT mice were i.d. grafted with B16F10 cells, and ears were treated with MC903 (on RE) or EtOH (on LE) as described in Figure 3A. Analyses of ELNs showed that MC903 treatment–induced GATA3^+^ Tregs were significantly reduced in *Crlf2*^CD11c–/–^ mice, in both frequency and cell number (Figure 4H), while GATA3^+^ Th2 cells exhibited a reduction in cell number but not in frequency (Figure 4H). These results suggest that TSLPR expressed by DCs was necessary for TSLP-driven GATA3^+^ Tregs in draining LNs.

### TSLP-promoted ST2^+^ Tregs suppress CD8^+^ T cell proliferation and IFN-γ production.

**Figure 5:**
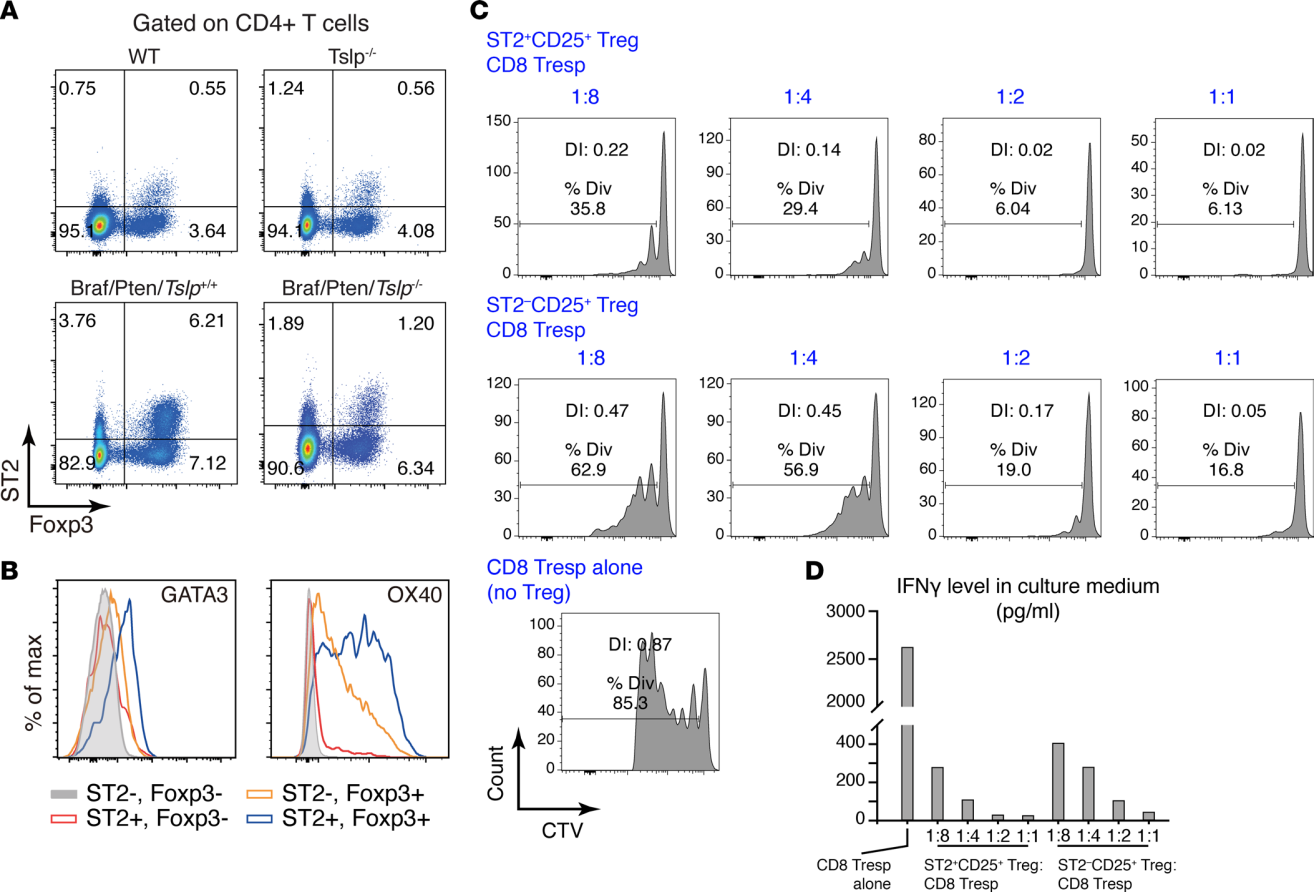
ST2^+^ Tregs suppress CD8^+^ T cell proliferation and their IFN-γ production. (**A**) Representative FACS plots of ST2 and Foxp3 among CD4^+^ T cells. (**B**) Histogram comparison for GATA3 and OX40 in the ST2/Foxp3 CD4^+^ T cell populations. (**C**) In vitro suppression of CD8^+^ T responder (CD8 Tresp) cell proliferation by ST2^+^ Tregs and ST2^–^ Tregs. CD8^+^ Tresp cells were labeled with CellTrace Violet (CTV) and stimulated with CD3/CD28 beads in the presence of ST2^+^ Tregs or ST2^−^ Tregs at different ratios (Treg/CD8^+^ Tresp at 1:1, 1:2, 1:4 or 1:8), or alone (no Treg). Percentage of division (Div) and division index (DI) are marked for each sample. (**D**) IFN-γ levels in the medium of the coculture of CD8^+^ Tresp and ST2^+^ Treg or ST2^−^ Treg, measured by ELISA. Each column represents 1 coculture. Data are representative of 4 independent experiments with similar results.

It has been recognized that Tregs are central players in downmodulating T cell–dependent immunity, particularly CD8^+^ cytotoxic T cell response and IFN-γ production in the context of tumors. We explored the regulatory role of GATA3^+^ Tregs using an in vitro functional assay (38). Because GATA3^+^ Tregs exhibited an ST2 signature, we first examined whether ST2 could be used as surrogate surface marker to sort these cells (as intracellular staining was needed for identifying GATA3). Gating CD4^+^ T cells with ST2 and Foxp3 (Figure 5A) showed that ST2^+^Foxp3^+^ (ST2^+^ Tregs) indeed exhibited GATA3 and OX40 expression compared with ST2^–^Foxp3^+^ (ST2^–^ Tregs) (Figure 5B). Moreover, the frequency of ST2^+^ Tregs was low in ILNs of WT and *Tslp*^–/–^ mice and was increased in Braf/Pten/*Tsl*p^+/+^ ILNs, but it was reduced in Braf/Pten/*Tslp*^–/–^ ILNs (Figure 5A), in a manner comparable with what was observed with GATA3^+^ Tregs (Figure 4B). Thus, ST2 was used as a surface marker to isolate GATA3^+^ Tregs in tumor-draining LNs.

The ST2^+^CD25^+^ Tregs and ST2^–^CD25^+^ Tregs cells were sorted from Braf/Pten ILNs and were cocultured with CD8^+^CD25^–^ T responders (CD8 Tresp) at different ratios from 1:8 to 1:1 (Treg/Tresp). Under these conditions, ST2^+^CD25^+^ Tregs suppressed CD8 Tresp proliferation and, strikingly, much more potently than ST2^–^CD25^+^ Tregs (Figure 5C). Moreover, IFN-γ produced by CD8 Tresp cells was reduced by coculturing with ST2^+^CD25^+^ Tregs or ST2^–^CD25^+^ Tregs in a ratio-dependent manner, with again ST2^+^CD25^+^ Tregs showing a stronger suppressive effect than ST2^–^CD25^+^ Tregs (Figure 5D). These results, thus, indicate that TSLP-promoted ST2^+^ Tregs exhibited a superior suppressive function on CD8^+^ T cell proliferation and IFN-γ production.

### TSLP-dependent accumulation of GATA3^+^ Tregs in melanoma tumor sites.

**Figure 6:**
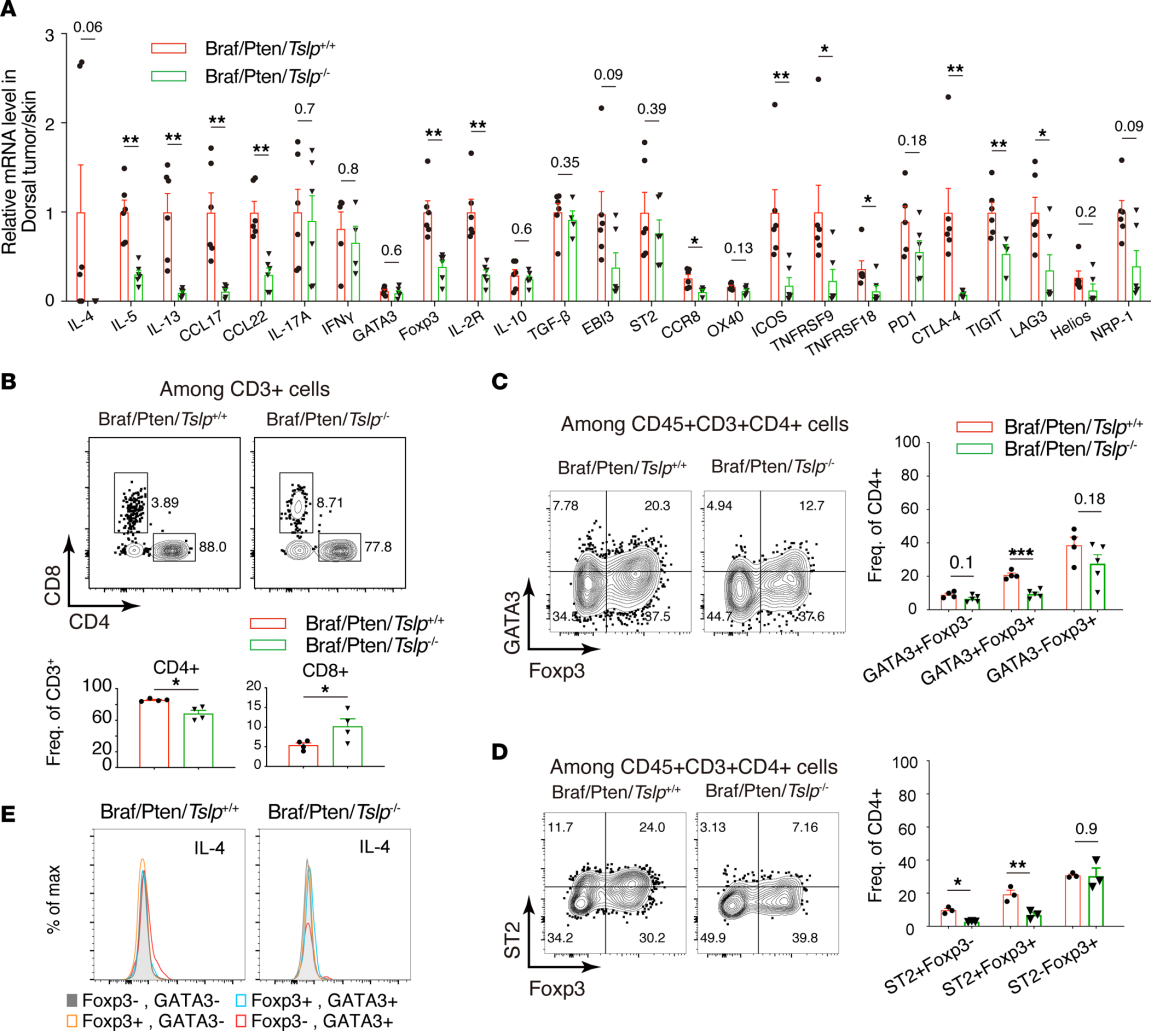
Absence of TSLP leads to reduced accumulation of GATA3^+^ Tregs in Braf/Pten tumors. (**A**) qPCR analyses of dorsal tumors from Braf/Pten/*Tslp*^+/+^ and Braf/Pten/*Tslp*^–/–^ mice. (**B**) Comparison of frequencies of CD4^+^ and CD8^+^ in CD3^+^ T cells in dorsal tumors from Braf/Pten/*Tslp*^+/+^ and Braf/Pten/*Tslp*^–/–^ mice. (**C** and **D**) Representative FACS plots and frequencies of GATA3/Foxp3 (**C**) or ST2/Foxp3 populations (**D**) among CD4^+^ T cells in dorsal tumors from Braf/Pten/*Tslp*^+/+^ and Braf/Pten/*Tslp*^–/–^ mice. (**E**) Histogram presentation of IL-4 in GATA3/Foxp3 populations among CD4^+^ T cells in dorsal tumors from Braf/Pten/*Tslp*^+/+^ and Braf/Pten/*Tslp*^–/–^ mice. Data are shown as mean ± SEM. Student’s *t* test. *n* ≥ 5 per group for **A**; *n* = 4 per group for **B**; *n* ≥ 3 per group for **C** and **D**. *P < 0.05; **P < 0.01; ***P < 0.001. Data are representative of 3 independent experiments with similar results.

We next examined whether GATA3^+^ Tregs accumulated at cutaneous melanoma sites in Braf/Pten mice and whether this was dependent on TSLP. qPCR analyses were performed with dorsal tumor samples comprising the melanoma tumors, together with the covering epidermis from Braf/Pten/*Tslp*^+/+^ and Braf/Pten/*Tslp*^–/–^ mice, or with dorsal skin from WT and *Tslp*^–/–^ mice. Compared with dorsal tumors from Braf/Pten/*Tslp*^+/+^, those from Braf/Pten/*Tslp*^–/–^ mice exhibited a reduction in Th2 cytokines IL-4, IL-5, and IL-13; chemokines CCL17 and CCL22; and Foxp3, IL-2Rα, CCR8, ICOS, TNFRSF9, TNFRSF18, CTLA-4, TIGIT, and LAG3, as well as a tendency for reduced ST2, EBI3, PD-1, and NRP-1. Hence, Braf/Pten/*Tslp*^–/–^ tumors exhibited reduced expression of these Th2- or Treg-associated genes (Figure 6A), as observed in tumor-draining LNs. Note that the comparison of RNA levels of these genes in the dorsal skin from WT and *Tslp*^–/–^ mice did not reveal any difference, suggesting that their expression in tumor-free skin was not impacted by the absence of TSLP (Supplemental Figure 9). Flow cytometry analyses of dorsal tumors showed that, among CD3^+^ T cells, the frequency of CD4^+^ cells was lower, whereas the frequency of CD8^+^ was higher in Braf/Pten/*Tslp*^–/–^ compared with Braf/Pten/*Tslp*^+/+^ tumors (Figure 6B). Among CD4^+^ T cells, Braf/Pten/*Tslp*^–/–^ mice exhibited a significant reduction of GATA3^+^ Tregs, as well as a tendency (although not significant) toward a reduction in GATA3^+^ Th2 cells (Figure 6C). Gating with ST2 and Foxp3 showed a similar pattern with a reduction in ST2^+^ Tregs in Braf/Pten/*Tslp*^–/–^ tumors (Figure 6D). Note also that, consistent with the results in draining LNs, the Th2 cytokine IL-4 was not detected in GATA3^+^ Tregs in cutaneous tumor sites from either Braf/Pten/*Tslp*^+/+^ or Braf/Pten/*Tslp*^–/–^ mice. Instead, it was detected in GATA3^+^ Th2 cells from Braf/Pten/*Tslp*^+/+^ tumors and reduced in Braf/Pten/*Tslp*^–/–^ tumors (Figure 6E). Together, these results indicate that TSLP was required for the accumulation of GATA3^+^ (or ST2^+^) Tregs at Braf/Pten cutaneous tumor sites.

We also examined whether more ST2^+^ Tregs accumulated in MC903-treated Braf mouse ears. Upon MC903 treatment, Braf/*Tslp*^+/+^ ears showed an increase in the frequency of ST2^+^ Tregs, which was diminished in Braf/*Tslp*^–/–^ ears (Supplemental Figure 10, A and B). In addition, MC903-induced accumulation of ST2^+^ Tregs was significantly reduced in B16F10-grafted *Crlf2*^CD11c–/–^ ears (Supplemental Figure 10, C and D). These results indicate that overexpression of TSLP promotes accumulation of ST2^+^ Tregs in tumor sites via signaling through TSLPR-expressing DCs.

### TSLP is overexpressed in the epidermis of human cutaneous primary melanoma.

**Figure 7:**
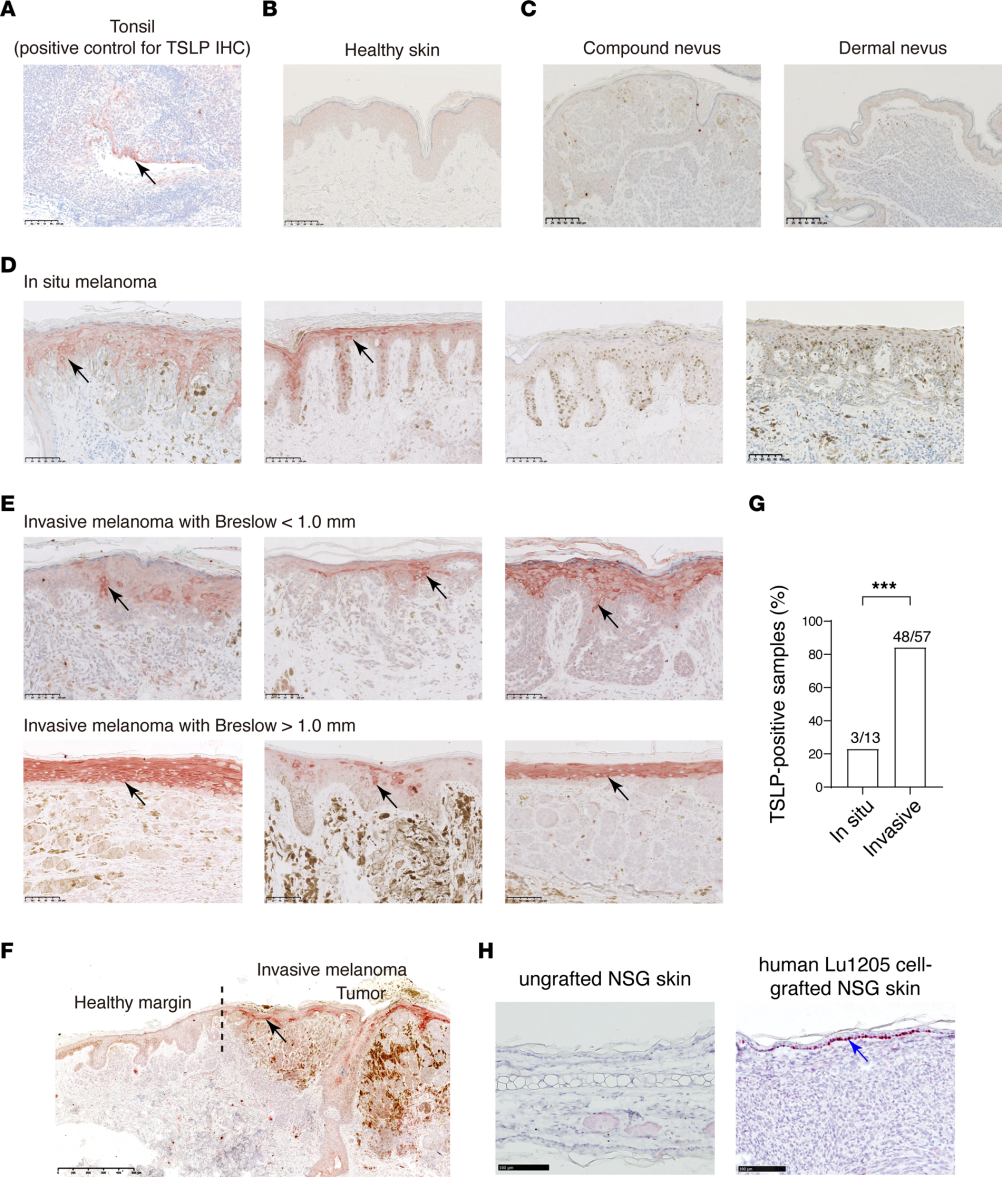
TSLP expression is upregulated in the epidermis of human primary melanoma. The expression of TSLP in human melanoma was analyzed by IHC staining with anti-TSLP antibody on FFPE sections. Representative TSLP IHC staining of tonsil (as positive control) (**A**), healthy skin (**B**), nevi (**C**), in situ melanoma (**D**), and invasive melanoma with Breslow < 1.0 mm or > 1.0 mm (**E**). (**F**) TSLP signal comparison of the tumor and the adjacent healthy margin of an invasive melanoma. The dotted line indicates the border of the tumor and the healthy margin. (**G**) Percentages of TSLP^+^ samples from the examined in situ melanoma or invasive melanoma biopsies. Two-tailed Fisher’s exact test; ***P < 0.001. (**H**) The intradermal (i.d.) grafting of human melanoma Lu1205 cells induces the expression of mouse TSLP in the epidermis of NSG mouse ears, shown by RNAScope ISH for TSLP. Blue arrow points to one of the positive signals in the epidermis. Scale bars: 100 μm (**A**–**D** and G); 500 μm (**F**).

To examine human TSLP expression in melanoma, biopsies from patients diagnosed with primary melanoma (*n* = 70, including 13 in situ melanoma and 57 invasive melanoma) were analyzed by IHC using a previously validated anti–human TSLP antibody (39). As positive CT, TSLP signals were confirmed in tonsil epithelial cells as previously reported (12, 40) (Figure 7A). TSLP was not detected in skin from healthy donors (Figure 7B), nor in junctional nevi (*n* = 2), compound nevi (*n* = 6), or intradermal nevi (*n* = 7) (Figure 7C and data not shown). In contrast, TSLP was detected in the epidermis in 3 of 13 in situ melanoma (23%) (2 TSLP^–^ and 2 TSLP^+^ biopsies are shown in Figure 7D) and in 48 of 57 invasive melanoma (85%) (6 biopsies are shown in Figure 7E) at varying levels, but all were located in epidermal suprabasal layers, similar to what was previously reported in atopic dermatitis lesioned skin (40). Therefore, there was a statistically significant increase in the percentage of TSLP-expressing samples in invasive melanoma compared with in situ melanoma (Figure 7G). However, there was no clear association observed between TSLP signals and Breslow depth, as TSLP was already strongly expressed even in some samples with Breslow < 1.0 mm (representative pictures from biopsies with Breslow depth < 1.0 mm or > 1.0 mm; Figure 7E). No signal for TSLP was seen in melanoma cells or in the dermis in any biopsies examined (Figure 7D and data not shown). In agreement with this, we did not detect TSLP expression in malignant cells, immune cells, or stromal cells in published single-cell RNA-Seq (scRNA-Seq) data sets for human cutaneous melanoma (41–43) (Supplemental Figure 11). Notably, these data sets did not include epidermal keratinocytes, reflecting a general disregard for the importance of these cells in previous studies on melanoma-associated TME.

The fact that TSLP^+^ signals were detected in epidermal keratinocytes overlying tumors, but not healthy epidermal borders (Figure 7F), suggests that tumor cells send signals that promoted epidermal TSLP production. Interestingly, i.d. grafting of human melanoma Lu1205 cells into NSG mouse ears induced the expression of mouse TSLP in the epidermis (Figure 7H). Thus, human melanoma cells, analogous to mouse melanoma cells, can possibly signal to keratinocyte to induce TSLP production in a conserved species cross-reactive manner.

### GATA3^+^ Tregs are enriched in human invasive melanoma.

**Figure 8:**
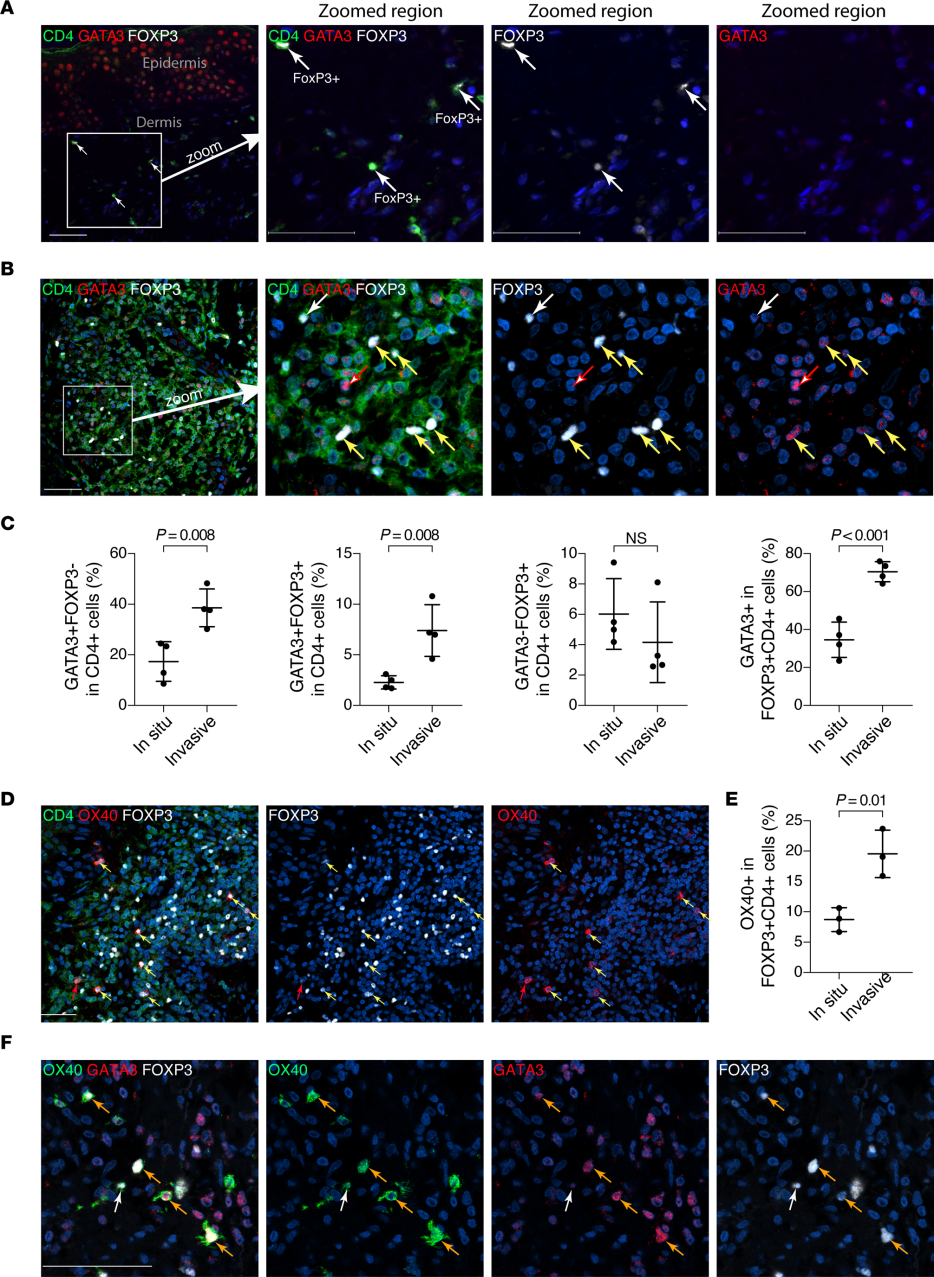
GATA3^+^Tregs are enriched in human invasive melanoma. (**A** and **B**) FFPE sections of healthy skin (**A**) or primary melanoma (**B**) were stained by multiplex IHC for CD4 (green), GATA3 (red) and FoxP3 (white), showing the detection of GATA3^+^FoxP3^–^ CD4 T cells (Th2; 1 of such cells is pointed by red arrow), GATA3^–^FoxP3^+^ CD4 T cells (GATA3^–^ Treg; 1 of such cells is pointed by white arrow), and GATA3^+^FoxP3^+^ CD4 T cells (GATA3^+^Treg; pointed by yellow arrows). (**C**) Comparison of invasive melanoma with in situ melanoma for percentages of GATA3^+^ Th2, GATA3^+^ Tregs, and GATA3^–^ Tregs in CD4^+^ T cells, as well as for percentages of GATA3^+^ cells within Tregs. (**D**) FFPE sections of primary melanoma were stained by multiplex IHC for CD4 (green), OX40 (red), and FoxP3 (white), showing that the majority of OX40^+^ cells are FoxP3^+^CD4^+^ Tregs. In this view, 7 OX40^+^FoxP3^+^CD4^+^cells are identified (pointed by yellow arrows), and 1 OX40^+^FoxP3^–^CD4^+^ cell is identified (pointed by red arrow). (**E**) Comparison of percentages of OX40^+^ in FoxP3^+^CD4^+^ Tregs between in situ and invasive melanoma biopsies. (**F**) FFPE sections of primary melanoma were stained by multiplex IHC for OX40(green), GATA3 (red), and FoxP3 (white), showing the detection of OX40 in GATA3^+^FoxP3^+^ Tregs (4 cells pointed by orange arrows) and GATA3^–^FoxP3^+^ Tregs (1 cell pointed by white arrow). Values in **C** and **E** are mean ± SEM. Student’s *t* test. *P* values are indicated. *n* = 4 (in **C**) and *n* = 3 (in **E**) per group. Scale bars: 50 μm. Data are representative of more than 3 independent multiplex IHC experiments with similar results.

We then investigated whether GATA3^+^ Tregs were present within the human primary melanoma TME. Analyses by multiplex immunofluorescence with antibodies against GATA3, FOXP3, and CD4 showed that, in healthy skin, GATA3 signals were detected in human epidermal keratinocytes as previously reported (44), but they were very rarely detected in dermal cells (Figure 8A; GATA3 in red), whereas a few FOXP3^+^ cells were detected in the dermis, all of which were CD4^+^ but were GATA3^–^ (Figure 8A). The dermis of healthy skin biopsies therefore, presented few GATA3^–^ Tregs but no detectable GATA3^+^ Tregs or GATA3^+^FOXP3^–^ cells.

In contrast, in invasive primary melanoma biopsies (Figure 8B), we observed many more CD4^+^ cells, among which we identified GATA3^+^FOXP3^–^ cells, GATA3^–^FOXP3^+^ cells, and GATA3^+^FOXP3^+^ cells. Among CD4^+^ T cells, the percentage of GATA3^+^FOXP3^–^ cells and the percentage of GATA3^+^FOXP3^+^ cells were higher in invasive melanoma compared with in situ melanoma, while the percentage of GATA3^–^FOXP3^+^ cells remained similar (Figure 8C). In an alternative comparison, among the FOXP3^+^CD4^+^ Tregs, the percentage of GATA3^+^ cells was elevated in invasive melanoma compared with in situ melanoma (Figure 8C). These results suggest that GATA3^+^ Tregs were enriched in primary melanoma, accumulating more in invasive melanoma than in situ melanoma.

Since OX40 represented a marker for GATA3^+^ Tregs in Braf/Pten mice (Figure 4E), we further examined whether this was the case for human melanoma. Note that literature reports on OX40 expression and signaling in human tumors remain largely controversial (45). We performed multiplex IHC with Abs against CD4, OX40, and FOXP3 showing that OX40 was predominantly expressed by CD4^+^ cells, and most OX40^+^ cells were FOXP3^+^CD4^+^ Tregs (Figure 8D). A higher percentage of OX40^+^ cells within FOXP3^+^CD4^+^ Tregs was observed in invasive melanoma compared with in situ melanoma, suggesting an enrichment of OX40^+^ Tregs in invasive melanoma (Figure 8E). Finally, we performed multiplex immunofluorescence with Abs against OX40, GATA3, and FOXP3, showing that OX40 was detected in both GATA3^+^FOXP3^+^ Tregs and GATA3^–^FOXP3^+^ Tregs, although it was not possible to differentiate the staining intensity (Figure 8F). These results, thus, indicate that, similar to what was observed in mouse primary melanoma, GATA3^+^ Tregs expressing OX40 were enriched in human melanoma.

### A Treg population in CD4^+^ T cells from human melanoma exhibits expression of GATA3 and other signature genes.

**Figure 9:**
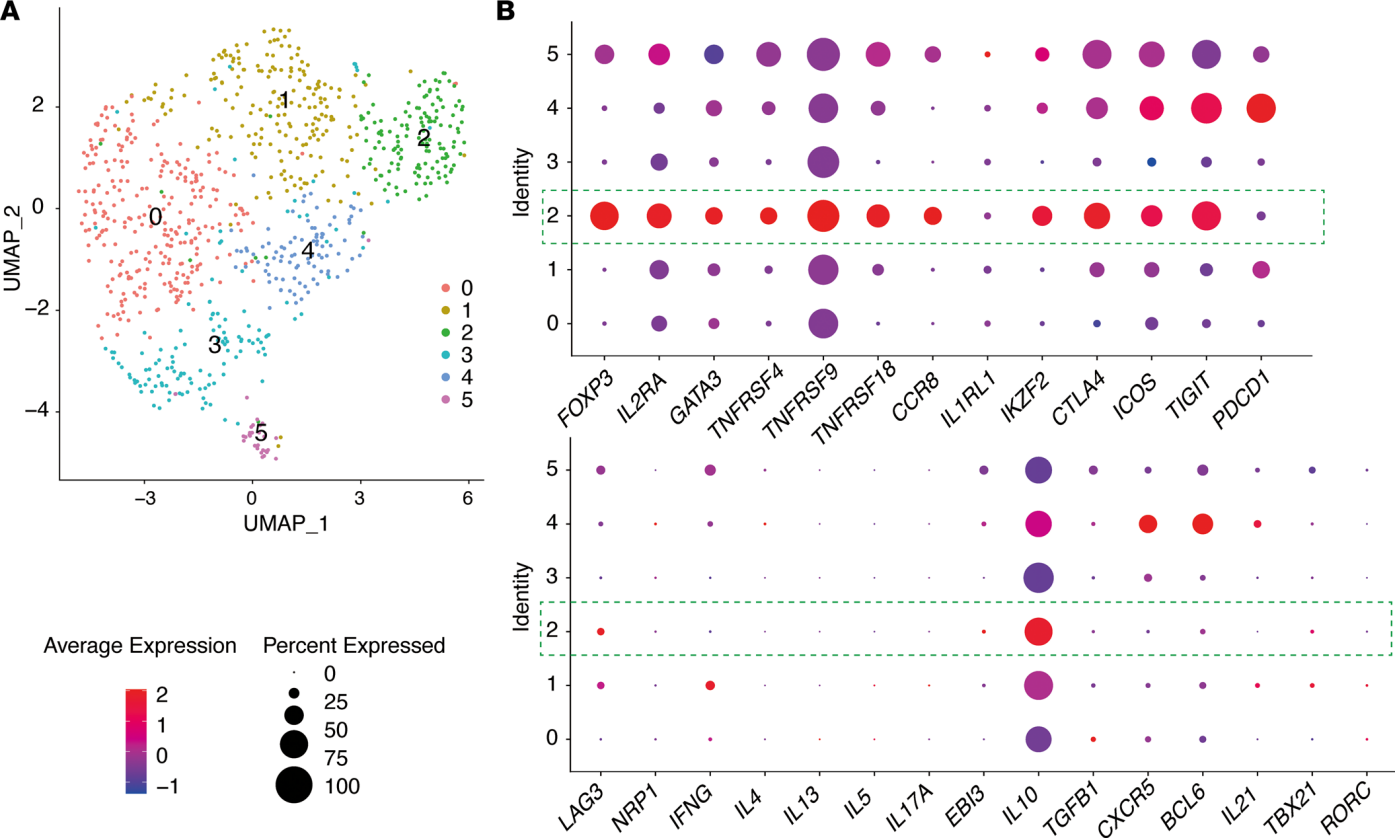
A Treg cluster identified in CD4^+^ T cells from human melanoma exhibits GATA3 and other signature gene expression. The 856 CD4^+^T cells from 33 human melanoma tumors were extracted from the processed data GSE115978, previously generated by scRNA-Seq study of Jerby-Arnon et al. (41), and analyzed with the Seurat pipeline. (**A**) Uniform manifold approximation and projection (UMAP) projection of single-cell transcriptomes colored by clusters (C0–C5). (**B**) Expression of the selected genes across different clusters is visualized with Dot Plot (the size of the dot encodes the percentage of cells within a class, while the color encodes the average expression level across all cells within a class; https://satijalab.org/seurat/reference/dotplot). C2 (highlighted by green dot lines) exhibited featured expression of FOXP3, IL-2RA, GATA3, TNFRSF4, TNFRSF9, TNFRSF18, CCR8, IKZF2, CTLA-4, ICOS, and TIGIT.

In a complementary approach to explore GATA3^+^ Tregs in human melanoma, we analyzed the scRNA-Seq data set reported by Jerby-Arnon et al. (41). The 856 CD4^+^ T cells identified by the authors from 33 human melanoma tumors were extracted and analyzed with Seurat pipeline. Unbiased graph clustering identified 6 clusters (C0–C5) of the CD4^+^ T cells (Figure 9A). Among them, C2 exhibited the featured Treg expression of *FOXP3* and *IL2RA*, with the highest expression among the 6 clusters for *GATA3*, *TNFRSF4* (encoding OX40), *TNFRSF9* (encoding 4-1BB), *TNFRSF18* (encoding GITR), *CCR8*, *IKZF2* (encoding Helios), *CTLA4*, *ICOS*, and *TIGIT* (Figure 9B and Supplemental Figure 12). These cells, thus, resemble the GATA3^+^ Tregs identified in mouse melanoma (Figure 4, A and D). We noticed, however, that expression of *IL1RL1* (encoding ST2), *NRP1*, and *LAG3* was very low, whereas although *PDCD1* (encoding PD-1) was expressed in C2, it was most strongly expressed in C4 and did not appear to be a signature gene for C2 (Figure 9B). C2 also did not show expression of *IL4*, *IL5*, *IL13*, *IFNG*, *IL17A*, or *TGFB1* but had the highest level of *IL10* (Figure 9B).

In addition, C5 exhibited the expression of Treg identity genes *IL2RA* and *FOXP3*, although it was weaker than C2, with a low or modest expression of *GATA3*, *TNFRSF4*, *TNFRSF9*, *CCR8*, *IKZF2*, *CTLA4*, *ICOS*, and *TIGIT*, suggesting that it may correspond to mouse GATA3^–^ Tregs. C4 showed an enriched expression of *CXCR5*, *BCL6*, *IL21, PDCD1*, *ICOS*, and *TIGIT*, suggesting that it may represent Tfh cells. None of the other clusters exhibited typical features for Th2 (*GATA3*, *IL4*, *IL13*) or Th17 (*RORC*, *IL17A*) cells, except that C1 showed a highest level for Th1 cytokine gene *IFNG* (Figure 9B and Supplemental Figure 12).

Together, these analyses of the single-cell expression of CD4^+^ T cells from human melanoma tumors provided evidence not only for the presence of Tregs with feature expression of *GATA3*, but also for their associated signature expression of *TNFRSF4* (OX40), *TNFRSF9*, *TNFRSF18*, *CCR8*, *IKZF2* (Helios), *CTLA4*, *ICOS*, and *TIGIT*.

## Discussion

**Figure 10:**
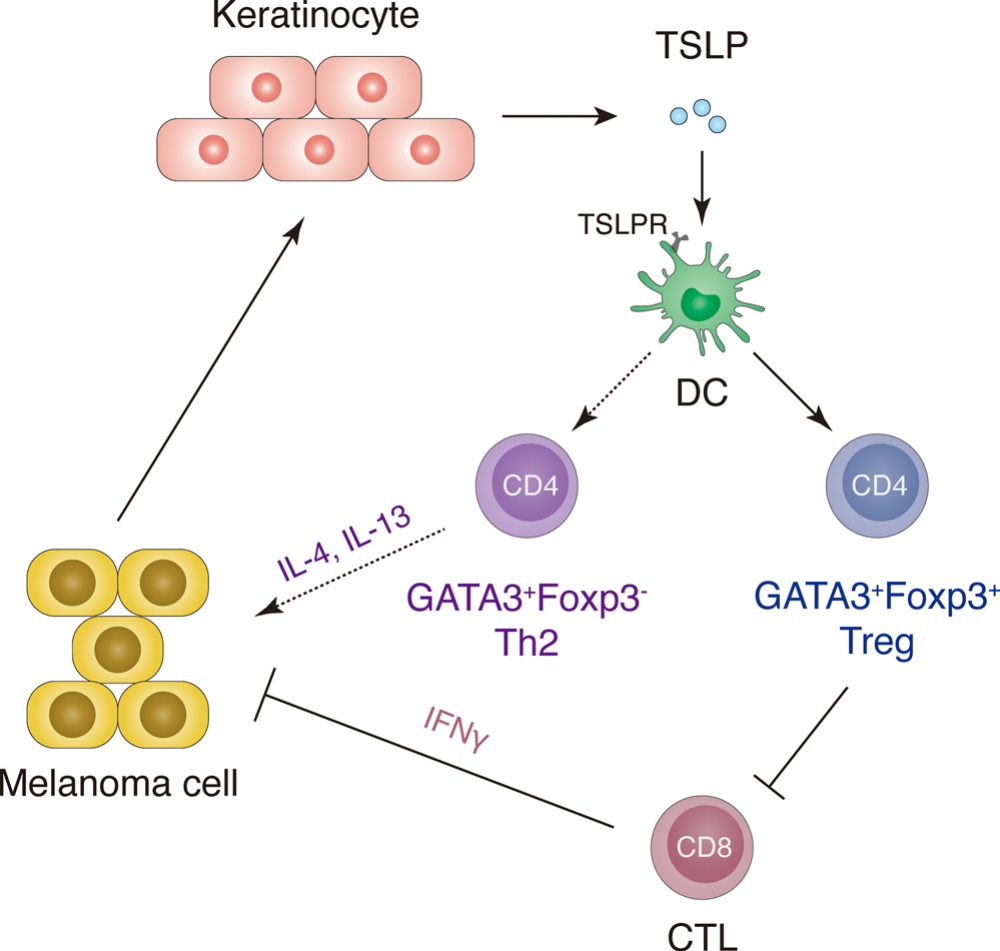
A schematic representation showing that skin TSLP plays an important role in a crosstalk of melanoma cells, keratinocytes, and immune cells that generates a tumor-promoting microenvironment in cutaneous melanoma. Signals derived from growing melanoma cells induce TSLP production by epidermal keratinocytes that subsequently act through TSLPR-expressing DCs to promote GATA3^+^Foxp3^+^ Tregs expressing a gene signature including ST2, CCR8, ICOS, PD-1, CTLA-4, and OX40 and exhibiting a superior activity in suppressing the proliferation and IFN-γ production of CD8^+^ cytotoxic T lymphocytes (CTL) cells. This TSLP-driven GATA3^+^Treg axis, in addition to the well-recognized TSLP-driven Th2 axis, may represent an underlying mechanism for the tumor-promoting role of TSLP in cutaneous melanoma.

Here, we report a protumoral role of TSLP derived from epidermal keratinocytes in driving the growth and metastasis of melanoma. Using mouse melanoma models or intradermal melanoma cell grafting combined with the ablation or overexpression of TSLP, we revealed a crosstalk between melanoma cells, keratinocytes, and immune cells in establishing a tumor promoting microenvironment. Keratinocyte-derived TSLP is induced by signals from melanoma tumor cells and subsequently acts through immune cells to promote melanoma growth, progression, and metastasis (Figure 10). Furthermore, we showed that TSLP signals through TSLPR-expressing DCs to play a previously unrecognized role in promoting GATA3-expressing Treg (GATA3^+^Foxp3^+^) cells expressing a signature of surface markers including ST2, CCR8, ICOS, PD-1, CTLA-4, and OX40 and exhibiting a potent suppressor activity on CD8^+^ cytotoxic T lymphocyte (CTL) proliferation and IFN-γ production. Together, our data define a potentially novel pathway by which TSLP promotes melanoma growth and metastasis via regulation of the tumor-associated immunosuppressive microenvironment (Figure 10).

Our study highlights the role of epidermal keratinocytes in modulating the cutaneous melanoma-associated microenvironment. This role has been poorly characterized, possibly due to the use of s.c. tumor cell grafting in mouse models that does not involve interactions with the epidermis and the fact that many analyses of human cutaneous melanoma often excluded the epidermal compartment. Moreover, TSLP has been controversially reported to play either a protumor or an antitumor role in various cancer types (reviewed in ref .11). Also, most of these previous studies focused on TSLP-Th2 mechanisms in cancer through the implication of Th2 cytokines IL-4 and IL-13 (10, 46) — as well as Th2-related cells, including basophils, eosinophils, and M2 macrophages (8, 47, 48) — whereas only a few reported a Th2-independent role of TSLP, such as on B cell precursors in lung metastasis (49). Here, we define a facet of the multiple potential functionalities of TSLP in modulating melanoma-associated immune responses, showing that keratinocyte-derived TSLP is crucial for the accumulation of Tregs — or, more specifically, GATA3^+^ Tregs — in both tumor-draining LNs and cutaneous tumor sites, reminiscent of “tumor-associated Treg” identified by several studies in various tumors in mice and humans (33–37). During the revision of this manuscript, Obata-Ninomiya et al. (50) reported a protumoral role of TSLP in colon cancer via a subset of Tregs coexpressing ST2, PD-1, and CTLA-4 that share certain similarities with GATA3^+^ Tregs that we reported here. Our data on melanoma are, therefore, complementary to those of Obata-Ninomiya et al. (50), suggesting that TSLP may have a rather broad protumoral role via modulation of such populations of Tregs. It is also noteworthy that, despite their GATA3 expression, we did not find any evidence that such Tregs express Th2 cytokines IL-4 or IL-13 either in mouse or human melanoma. This is in contrast to the “Th2-like Tregs” recently reported in melanoma (51) that were shown to produce more Th2 cytokines than the other Tregs when activated in vitro with anti-CD3/CD28 beads. This discrepancy possibly reflects the difference of in vivo versus in vitro context, that the level of Th2 cytokine expression by GATA3^+^ Tregs is much lower than GATA3^+^ Th2 cells, or the expression of Th2 cytokines by GATA3+ Tregs is induced only at a specific stage or upon certain types of stimulation.

We show that TSLP-promoted GATA3^+^ (ST2^+^) Tregs exhibit a highly suppressive activity for CD8^+^ CTLs, representing a plausible pathway for the protumoral function of TSLP. In agreement with this, a higher CD8/CD4 ratio was observed in Braf/Pten tumors following TSLP ablation (Figure 6B). However, it should be noted that a wide variety of cell types have been described as direct targets of Treg-mediated suppression, involving different pathways (52). In fact, we show that TSLP controlled not only the number of GATA3^+^ Tregs, but also their expression of receptors ST2 and CCR8 and a number of inhibitory checkpoints, including ICOS, CTLA-4, and PD-1 (Figure 4E). Thus TSLP-promoted GATA3^+^ Tregs may also exert their immunosuppressive function through their crosstalk with tumor-associated DCs (53) or myeloid-derived suppressor cells (MDSCs) (54). Notably, we observed that Braf/Pten/*Tslp*^–/–^ tumors exhibited a reduced accumulation of CD11b^+^Ly6C^hi^Ly6G^–^ myeloid cells (monocytic M-MDSCs) and CD11b^+^Ly6C^int^Ly6G^+^ myeloid cells (PMN-MDSCs) compared with Braf/Pten mice, accompanied by reduced serum levels of S100A8/A9 (Supplemental Figure 13). Recently, it was shown that MDSCs drive expansion of Tregs via secretion of S100A8/A9 (55). However in our model, the differences concerning MDSCs between Braf/Pten and Braf/Pten/*Tslp*^–/–^ were only observed at a later stage compared with the differences on GATA3^+^ Tregs (Supplemental Figure 13). This suggests that MDSCs and S100A8/A9 may rather be downstream of GATA3^+^ Tregs. Nevertheless, the function of GATA3^+^ Tregs in melanomagenesis remains to be better defined using strategies to selectively deplete this Treg population — for example, by CCR8 antibody–mediated depletion as reported recently (56).

Our data provide evidence that TSLP signals through TSLPR-expressing DCs to promote GATA3^+^ Treg generation/expansion. Interestingly, it has been reported that skin-derived TSLP promoted Treg expansion through DCs (57) and that TSLP-conditioned DC-induced Tregs (58), although these previous studies did not examine whether the Tregs are a specified subset as shown here. On the other hand, TSLP has been recognized to induce Th2 and Tfh responses via DCs in mice (20, 27) and humans (28, 29). However, it is unclear whether the same or different DC types are implicated in GATA3^+^ Treg induction/expansion versus Th2 or Tfh induction, which signaling pathways are involved, and what eventual crosstalk exists between these axes. Nevertheless, the DC-mediated mechanism for the TSLP/GATA3^+^(ST2^+^) Treg axis reported here differs from that reported by Obata-Ninomiya et al. (50), who show that TSLP signals directly via TSLPR predominantly expressed by ST2^+^ Tregs in mouse and human colon cancer. Indeed, examination of CRLF2 gene expression in CD4^+^ T cells from human melanoma scRNA-seq data did not reveal higher expression of CRLF2 by GATA3^+^ Tregs compared with other CD4^+^ T cells (Supplemental Figure 11B). Together, our findings illustrate differences in the mechanism of action of TSLP in cutaneous melanoma and in colon cancer. Also, as DCs were not sufficiently represented in the available human cutaneous melanoma data sets (Supplemental Figure 11A), further studies will be required to investigate TSLPR-expressing DCs and their association with TSLP-induced GATA3^+^ Tregs in human melanoma.

Our data suggest that targeting TSLP may constitute a new strategy to modulate the melanoma-associated immune microenvironment either alone or in combination with other therapeutic approaches. Indeed, we evaluated anti–PD-1 blockade in Braf/Pten and Braf/Pten/*Tslp*^–/–^ mice (Supplemental Figure 14). No clear effect on Braf/Pten tumor growth was observed, although a mild tendency for a further delay in tumor growth in Braf/Pten/*Tslp*^–/–^ mice (Supplemental Figure 14) was seen. These observations may reflect the fact that the effect of anti–PD-1 is model dependent, with the Braf/Pten model being rather insensitive. In addition, TSLP-driven GATA3^+^ Tregs express high levels of PD-1, with TSLP ablation leading to diminished PD-1, perhaps explaining why there was no further clear synergistic effect of PD-1 blockade. Nevertheless, further studies involving TSLP/TSLPR blockade combined with therapeutic options such as MAP kinase inhibitors (targeting tumor cells) or immune checkpoint therapies (targeting immune cells) are required. Of particular note, the recent approval of tezepelumab (anti-TSLP antibody) to treat asthma in humans provides the possibility to translate our mouse studies to human melanoma. Moreover, it will be important to assess whether TSLP expression or/and serum levels could serve as prognostic factors for cutaneous melanoma patients, as recently reported in gastric cancer (59), oropharyngeal squamous cell carcinoma, or epithelial ovarian carcinoma (60, 61).

Lastly, it will be important to determine how melanoma cells signal to induce keratinocyte TSLP expression. While we show that TSLP expression by keratinocytes is likely induced by signals derived from tumor cells, the molecules involved remain to be determined. Cancer cell–produced IL-1β was previously shown to induce TSLP in breast or pancreatic cancers (8, 62, 63), but ex vivo culture of epidermis from WT mice with IL-1β did not induce TSLP production (Supplemental Figure 15), suggesting that, at least on its own, IL-1β does not induce the expression of epidermal TSLP. Further exploration of TSLP induction signals in tumor contexts, including recently reported tumor-derived extracellular vesicles (64), is needed to identify new strategies for the modulating inflammatory microenvironment associated with cutaneous melanoma.

## Methods

Supplemental Methods are available online with this article.

### Animals.

Mice bearing the *Tyr:Cre-*ER^T2^ transgene (65), *Braf*^LSL–V600E/+^ (16), floxed allele of *Pten* (22), and *Tslp^–/–^* (19) were as described. CD11c-Cre^Tg/0^ mice (30) were from The Jackson Laboratory (stock no. 008068). All mice were bred to C57BL/6J background for at least 9 generations and were bred to generate experimental mice with the desired genotypes. WT C57BL/6J and NSG mice were from The Jackson Laboratory. Mice bearing the conditional allele of *Crlf2* were generated by us at the ICS (for details, see Supplemental Methods).

### Human biopsies.

Sections (5 μm) from formalin-fixed paraffin-embedded (FFPE) tissue from primary melanoma and nevi were retrospectively retrieved from the department of dermatology of Strasbourg University Hospital (for details, see Supplemental Methods).

### Mouse primary melanoma induction.

Mouse primary Braf or Braf/Pten melanoma was induced either by i.p. injection of Tam or topical application of 4-HT. For details, see Supplemental Methods. Dorsal solid tumors were measured for their length (L) and width (W) using Digimatic Caliper (Mitutoyo), and tumor size was calculated using the ellipsoid volume formula (1/2 × L × W^2^) (66).

### Melanoma cell grafting.

Mouse melanoma B16F10 cell line was provided by Lionel Larue (Institut Curie, Paris, France) B16F10 cells (1 × 10^4^ cells) were i.d. injected into mouse ears. Tumor growth was monitored by digital photography of the skin and analyzed using ImageJ (NIH) software. The Lu1205 cell line derived from human melanoma patients (2 × 10^4^) was i.d. injected into ears of NSG mice.

### MC903 topical application.

MC903 (Calcipotriol, MilliporeSigma, C4369) was dissolved in 100% ethanol and topically applied on mouse ears (2 nmol in 25 μL) as described before (19). For the dose-dependent experiments (Figure 2, F and G), we applied 0.1 nmol, 0.4 nmol, or 2 nmol of MC903.

### Statistics.

Data were analyzed using GraphPad Prism by Student’s 2-tailed unpaired *t* test with Welch’s correction or the 2-tailed Mann-Whitney *U* rank-sum nonparametric test, depending on results from the Kolmogorov-Smirnov test for normality. Comparison of more than 2 groups was performed by ordinary 1-way ANOVA, followed by Tukey’s post hoc test. Data are presented as mean ± SEM (for Student’s *t* test or 1-way ANOVA), or with median (for Mann-Whitney *U* rank-sum nonparametric test). Two-tailed Fisher’s exact test was used to compare percentages of TSLP^+^ samples from the examined in situ melanoma or invasive melanoma biopsies (see Figure 7). *P* < 0.05 was considered to be statistically significant.

### Study approval.

Breeding and maintenance of mice were performed under IGBMC institutional guidelines, and animal experiments were performed in accordance with recommendations of the European Community (86/609/EEC) and Union (2010/63/UE) and the French National Committee (87/848) guidelines and policies, and with approval of the National Ethics Committee.

## Author contributions

WY, BG, DC, AB, ID, and ML conceived and designed the study. WY BG, and DC conducted most of experiments and acquired data, and the chronological order of their contribution to this work was used in assigning the authorship order among co–first authors. AB contributed to IHC and multiplex IF studies with human melanoma biopsies. DC and P. Meyer performed in vitro Treg functional study. DC, P. Meyer, and GD performed scRNA-Seq data analyses. CH, P. Meyer, P. Marschall, EF, JS, and MG contributed to flow cytometry analyses. CH, EF, and PH contributed to qPCR analyses. PL contributed to IHC analyses of mouse melanoma samples. GM initiated and contributed to Braf/TSLP and Braf/Pten/TSLP mouse breeding and cohort generation. MCB contributed to design and generation of *Crlf2*-floxed mice. EF performed ex vivo culture study and prepared mouse cohorts. AB and DL provided human melanoma FFPE biopsies and analyzed IHC data. WY, BG, DC, AB, LD, ID, and ML analyzed and interpreted data. WY, BG, DC, AB, DL, ID, and ML wrote and revised the manuscript. ML directed the study and supervised the work.

## Supplementary Material

Supplemental data

## Version 1 09/15/2022

In-Press Preview

## Version 2 11/08/2022

Electronic publication
