# Supplementary material for: Keratinocyte-derived cytokine TSLP promotes growth and metastasis of melanoma by regulating the tumor-associated immune microenvironment
Source: JCI Insight. 2022 Nov 8;7(21):e161438. doi: 10.1172/jci.insight.161438 (PMC9675576; doi:10.1172/jci.insight.161438)
Supplement: Supplemental data [file jciinsight-7-161438-s214.pdf]

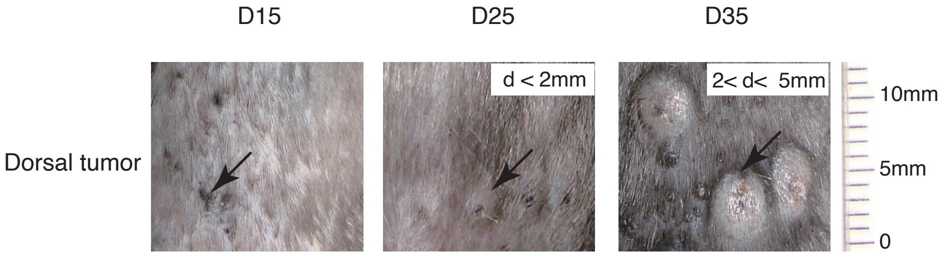

Supplemental Figure 1. Appearance of dorsal tumors in Braf/Pten mice following the injection of Tamoxifen. Eight to 12 weeks old *Tyr::Cre*$^{ERT2(tg/0)}$*::* *Braf*$^{LSL\text{-}V600E/+}$*: Pten*$^{lox/lox}$ mice were injected intraperitoneally (i.p.) with tamoxifen for 2 consecutive days (Braf/Pten mice). Dorsal tumor appearance is shown at the indicated days after tamoxifen injection.

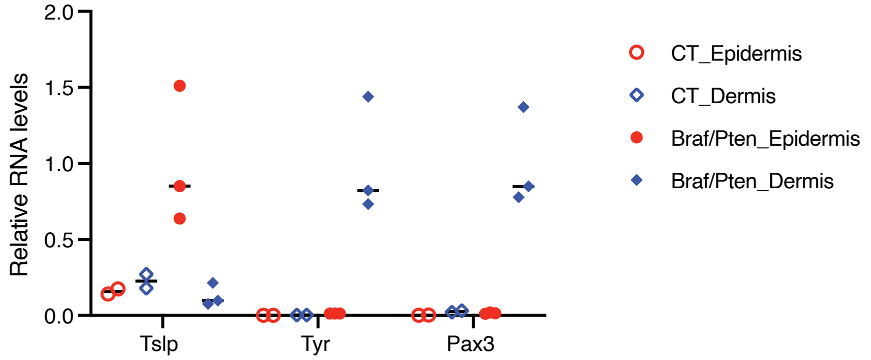

Supplemental Figure 2. TSLP is predominantly induced in the epidermal compartment of cutaneous melanoma of Braf/Pten mice. Ears from control (CT) or Braf/Pten mice (at D40 following 4-HT treatment) were digested with Dispase (4 mg/ml) to separate the epidermis from the dermis. RNA was extracted and RT-qPCR analyses showed that TSLP was induced in the epidermal (but not dermal) compartment of Braf/Pten mice. In contrast, melanoma cell-specific genes Tyrosinase (Tyr) and Paired box 3 (Pax3) were strongly detected in the dermal compartment of Braf/Pten mice compared with CT mice. Median is shown for each group.

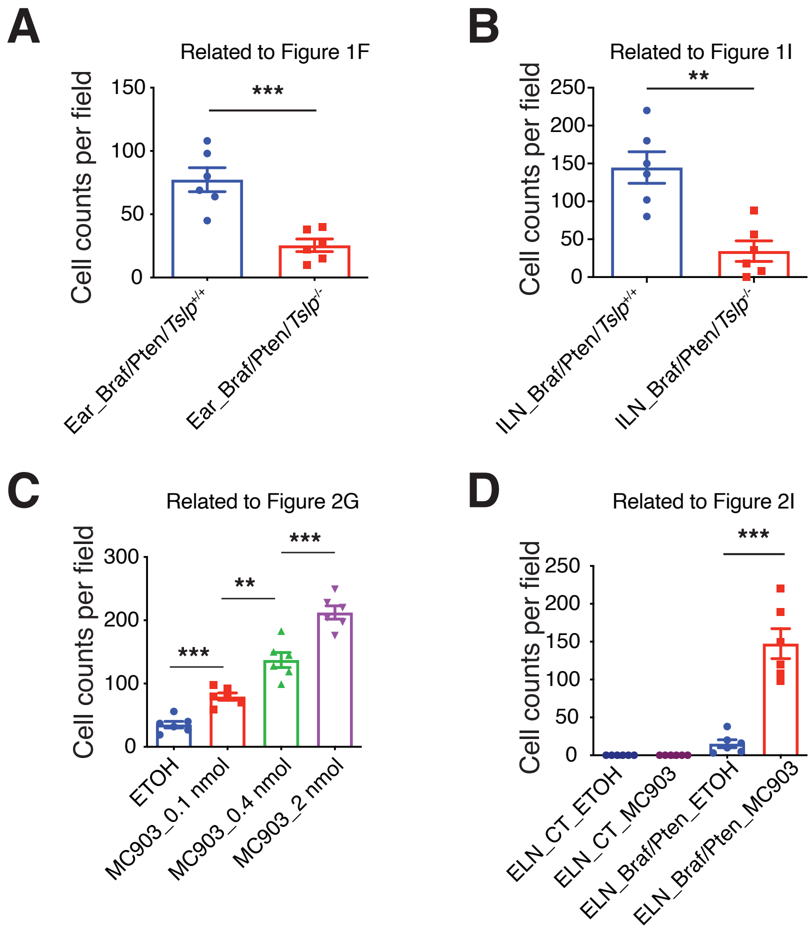

Supplemental Figure 3. Summary of counts for Sox10⁺ cells from immunostained paraffin sections. (**A**) ear sections related to Figure 1F. (**B**) Inguinal lymph node (ILN) sections related to Figure 1I. (**C**) ears sections related to Figure 2G. (**D**) Ear-draining lymph nodes (ELN) sections related to Figure 2I. One point corresponds to the average of $Sox10^{+}$ cells of 5 microscopic fields (magnification x20) of one section. Values are mean ± SEM (n=6 for each group). Students' t-test. **, $p<0.01$; ***, $p<0.001$.

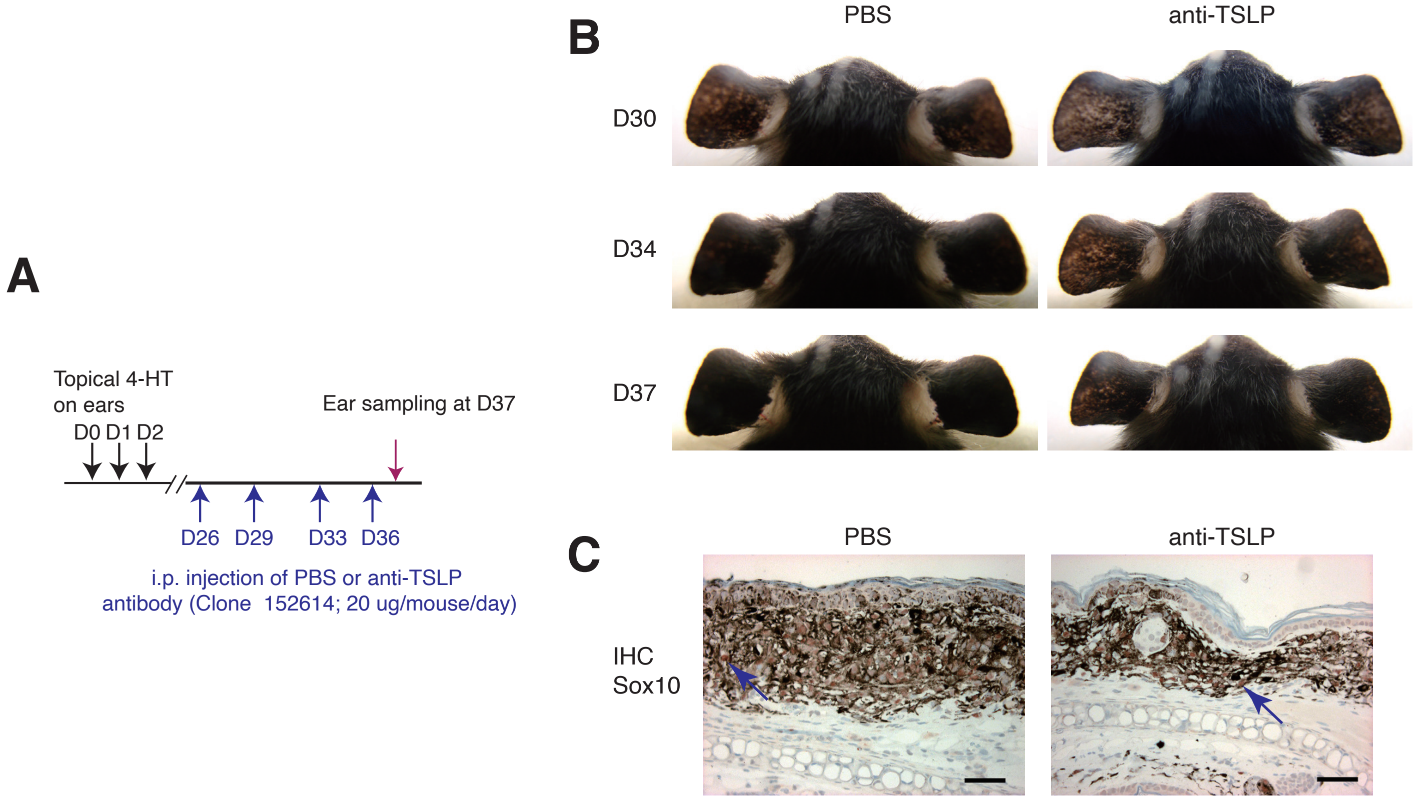

Supplemental Figure 4. Anti-TSLP blockade retards the formation of pigmentated lesions in the ears of Braf/Pten mice. (**A**) Experimental protocol. Ears of Braf/Pten mice were topical treated with 4-hydroxytamoxifen (4-HT) for 3 days (D0-D2). Starting from D26, mice were i.p. injected with PBS or with anti-TSLP neutralization antibody (Clone 152614, R&D; 20 µg/mouse/injection. Ref: You et al 2016 Plos One; PMID: 27467143; Cipolat et al. 2014 Elife; PMID: 24843010) 2 times per week for 2 wks. (**B**) Ear appearance at D30, D34 and D37. Photos are representive for 5 Braf/Pten mice treated with PBS, and 6 Braf/Pten mice treated with anti-TSLP antibody. (**C**) IHC of SOX10 on ear sections at D37. Blue arrows point to one of the positive signals (in dark red). Bar = 50 µm.

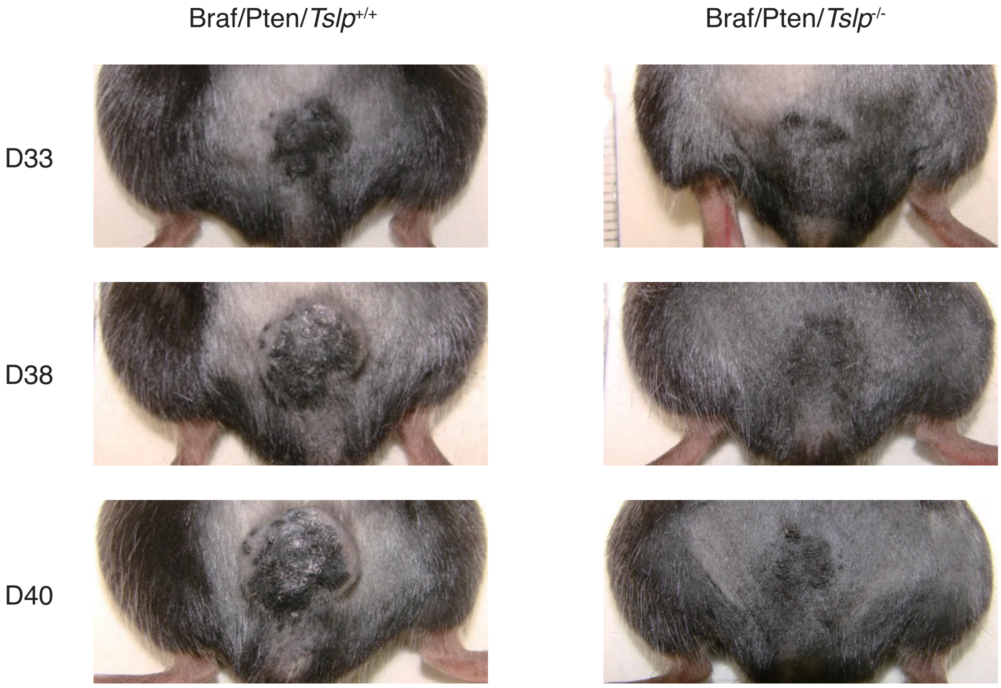

Supplemental Figure 5. Appearance of dorsal tumors in a Braf/Pten/*Tslp*$^{+/+}$ mouse and a Braf/Pten/*Tslp*$^{-/-}$ mouse at the indicated time points following topical 4-hydroxytamoxifen (4-HT) treatments.

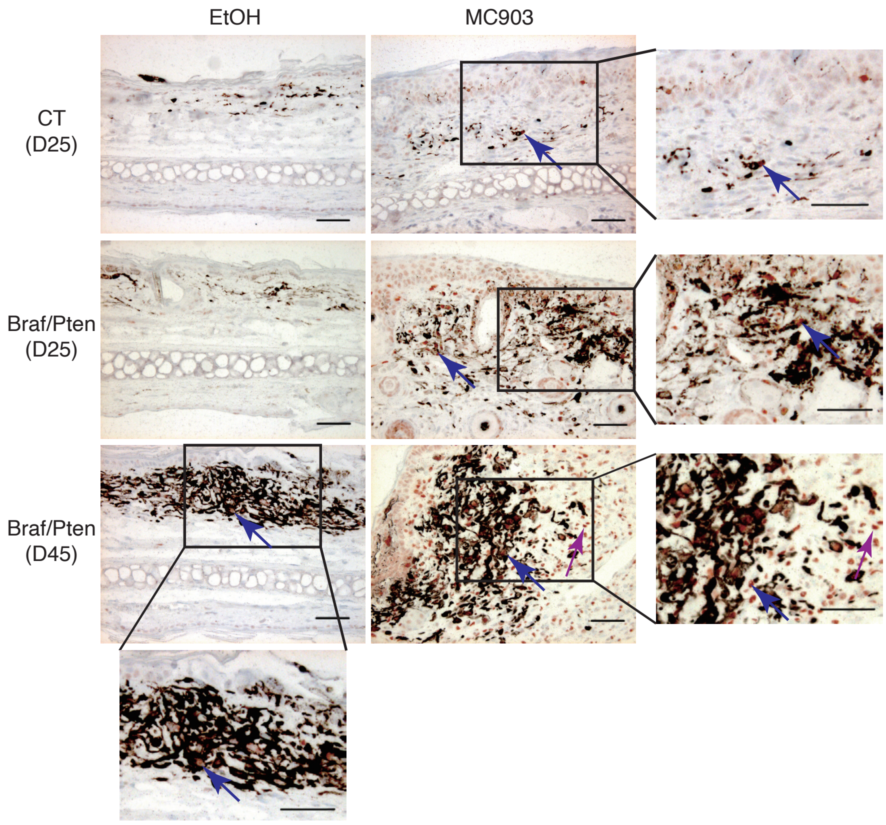

Supplemental Figure 6. IHC staining of Sox10 on ear sections from ethanol- and MC903-treated CT and Braf/Pten mice at D25 and D45. Blue arrows point to one of the positive cells (in dark red) inside the pigmented region and violet arrows point to one of the positive cells within hypo-pigmented region. Bar = 50 µm for all pictures.

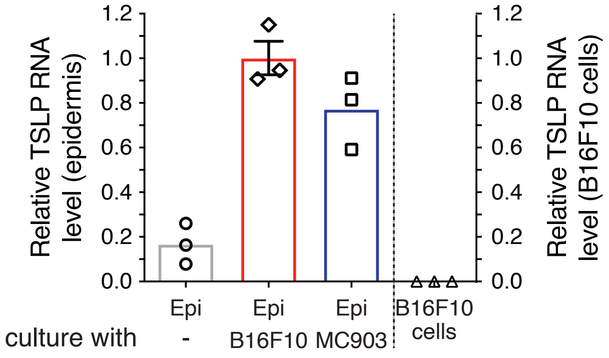

Supplemental Figure 7. TSLP RNA level is induced ex vivo in epidermis co-cultured with B16F10 melanoma cells. The epidermis prepared from ears of wild-type (WT) mice was floated on the medium of plated 80% confluent B16F10 cells. After 24 hrs co-culture, the epidermis was harvested for RNA analyses. The epidermis cultured alone, or in the presence of MC903 (10 μM), as positive control for the induction of TSLP, was analysed in parallele. Note that TSLP RNA level was undetectable in B16F10 cells. Values are mean ± SEM (n = 3). Data are representative of 2 independent experiments with similar results.

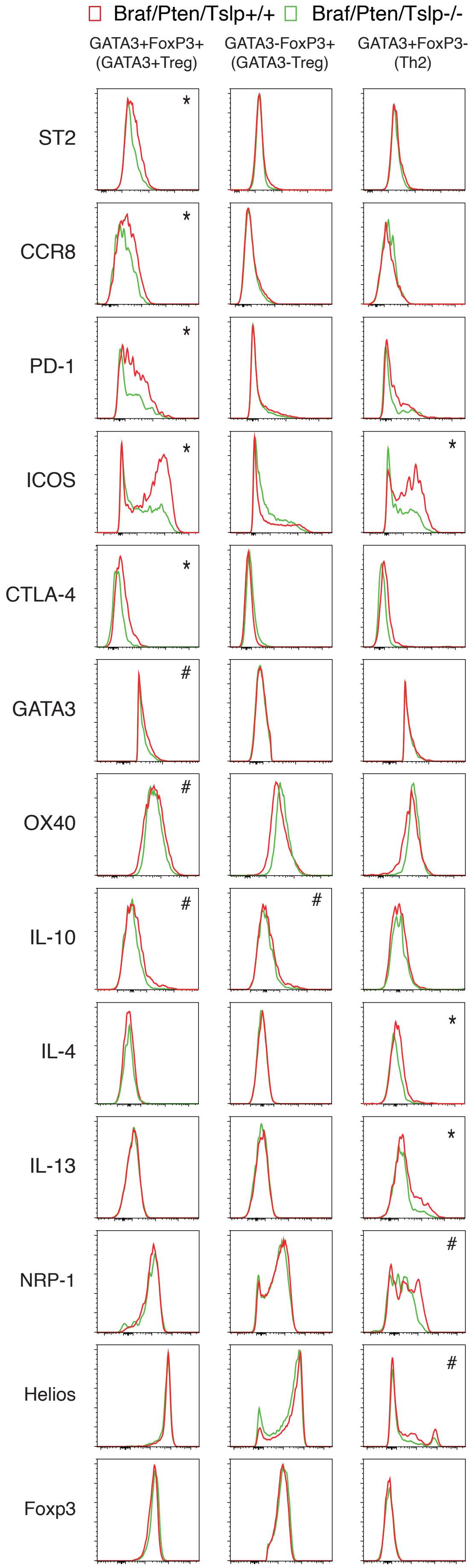

Supplemental Figure 8. Histogram comparison of indicated markers in GATA3$^+$FoxP3$^+$ (GATA3$^+$ Treg), GATA3$^-$FoxP3$^+$ (GATA3$^-$ Treg) and GATA3$^+$FoxP3$^-$ (Th2) CD4 T cells, between Braf/Pten/*Tslp*$^{+/+}$ and Braf/Pten/*Tslp*$^{-/-}$ mouse tumor-draining lymph nodes. *, median fluorescent intensity (MFI) is significantly lower in Braf/Pten/*Tslp*$^{-/-}$ compared to Braf/Pten/*Tslp*$^{+/+}$; #, MFI tends to be lower in Braf/Pten/*Tslp*$^{-/-}$ compared to Braf/Pten/*Tslp*$^{+/+}$.

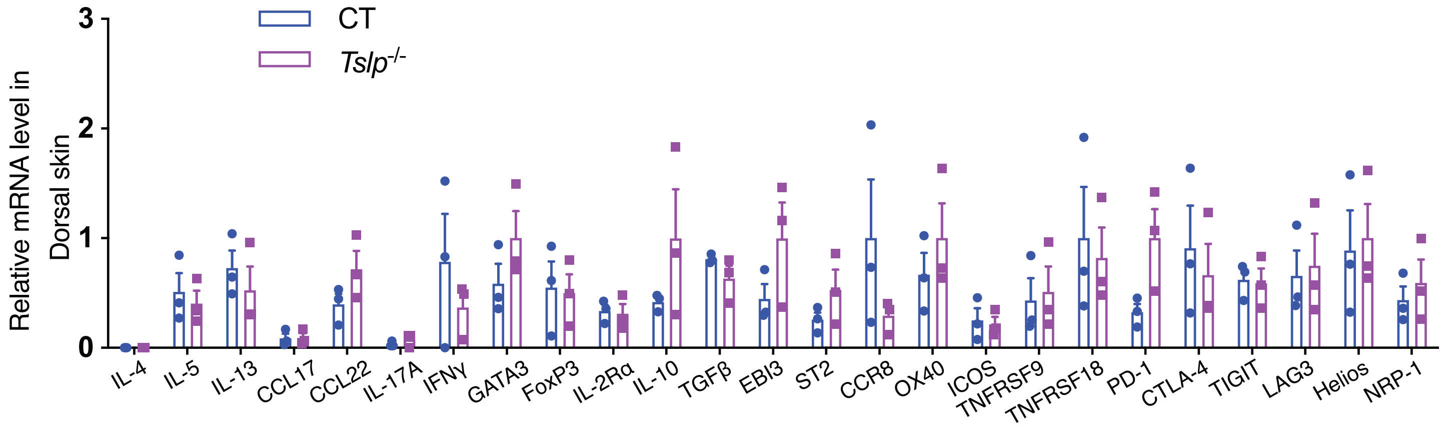

Supplemental Figure 9. Comparison of RNA levels of indicated genes in dorsal skin from WT and *Tslp*$^{-/-}$ mice. Values shown are mean ± SEM (n=3 per group). Data are representative of three independent expeirments with similar results.

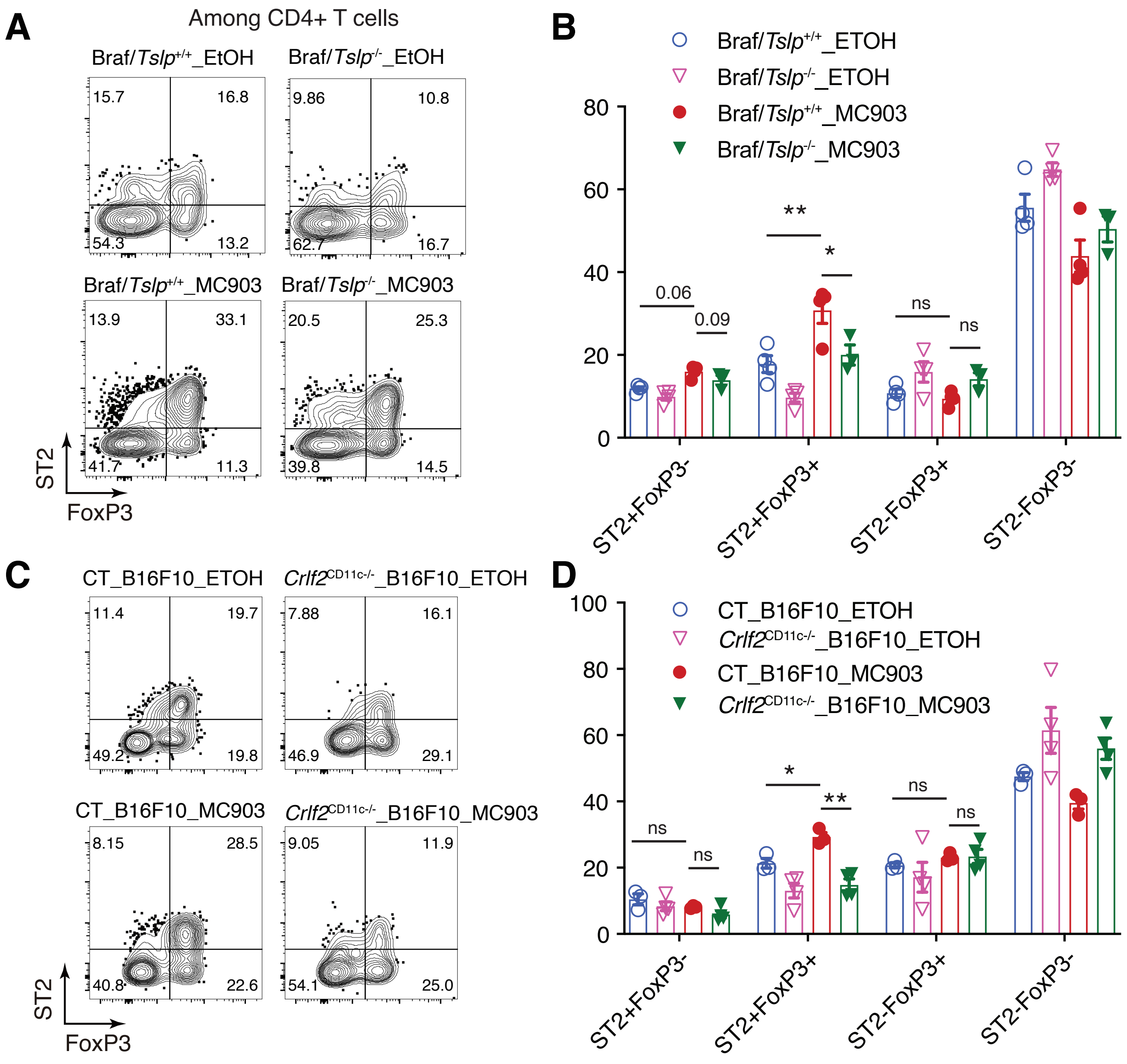

Supplemental Figure 10. Overexpression of TSLP promotes ST2⁺ Tregs in Braf or B16F10-grafted ears. (**A-B**) Representative FACS plots (**A**) and frequency (**B**) of ST2/FoxP3 populations among CD4⁺ T cells in ears from ETOH- or MC903-treated Braf/*Tslp*$^{+/+}$ and Braf/*Tslp*$^{-/-}$ mice. (**C-D**) Representative FACS plots (**C**) and frequencies (**D**) of ST2/FoxP3 populations among CD4⁺ T cells in ears from ETOH- or MC903-treated CT (control, CD11c-Cre$^{0/0}$/*Crlf2*$^{L2/L2}$) and *Crlf2*$^{CD11c-/-}$ (CD11c-Cre$^{Tg/0}$/*Crlf2*$^{L2/L2}$) mice, showing that TSLPR expressed by DCs is required for the induction of ST2⁺ Tregs by MC903 in B16F10-grafted ears. Values are mean ± SEM. One-way ANOVA test. For **B**, n=4 for all groups except n=3 for Braf/*Tslp*$^{-/-}$_MC903 groups; for **D**, n=3 for CT groups and n=4 for *Crlf2*$^{CD11c-/-}$ group. *, $p<0.05$; **, $p<0.01$. ns, non-significant. Data are representative of 3 independent experiments with similar results.

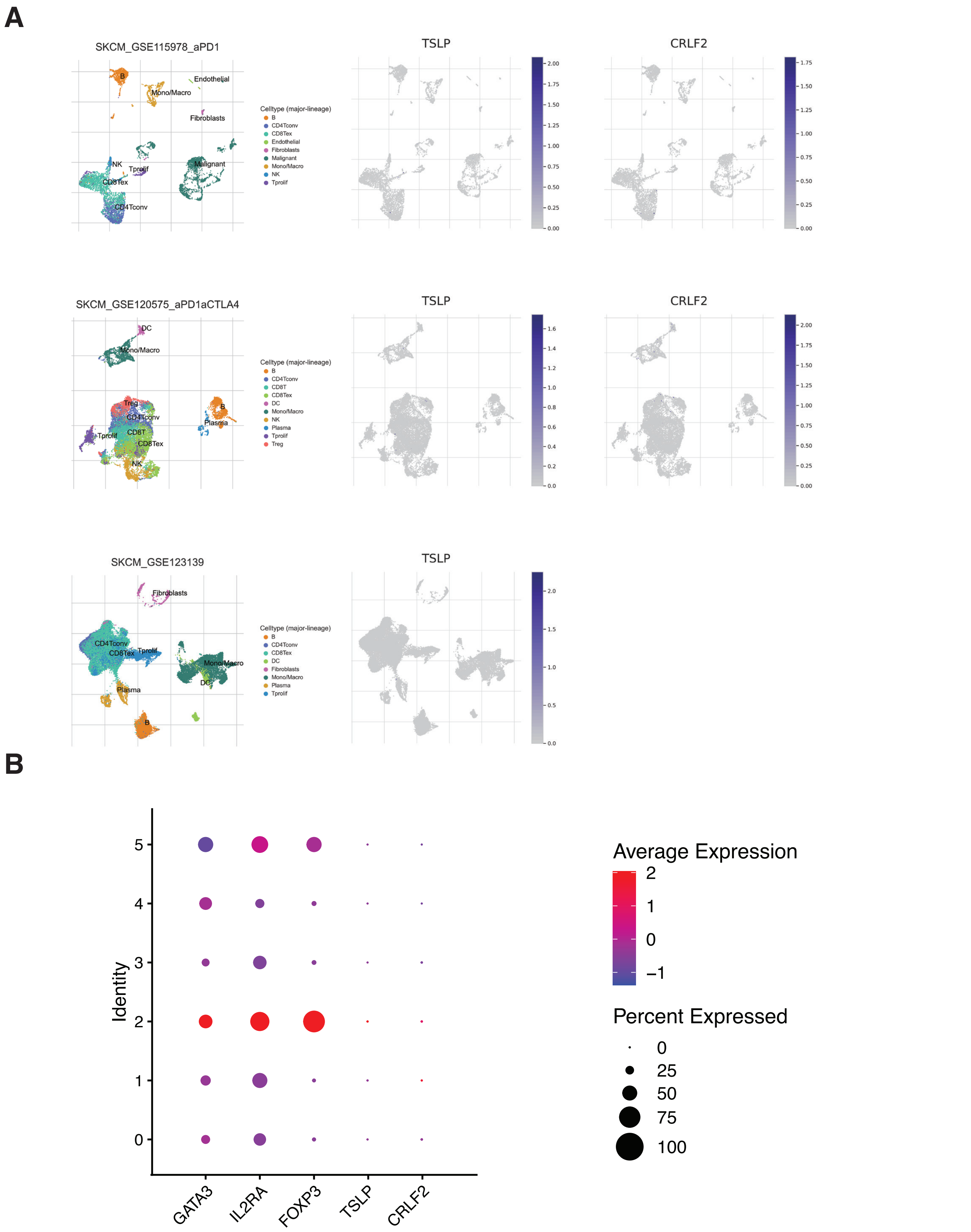

Supplemental Figure 11. (**A**) Cell-type annotations and featured plots for TSLP and CRLF2 gene expression in human skin cutanous melanoma datasets GSE115978, GSE120575, and GSE123139 analyzed with scRNAseq web resource Tumor ImmuneSingle Cell Hub (TISCH,http://tisch.comp-genomics.org; Sun et al. 2021 Nucleic Acids Res. PMID: 33179754). Note that CRLF2 gene was not annotated in GSE123139. (**B**) Expression of TSLP and CRLF2 across different clusters identified in $CD4^+$ T cells from human melanoma tumor dataset GSE115978. As shown in Figure 9, Cluster 2 (C2) is identified as GATA3-expressing Treg. Data show that C2 does not exhibit a higher expression of CRLF2 compared with other $CD4^+$ T cell clusters.

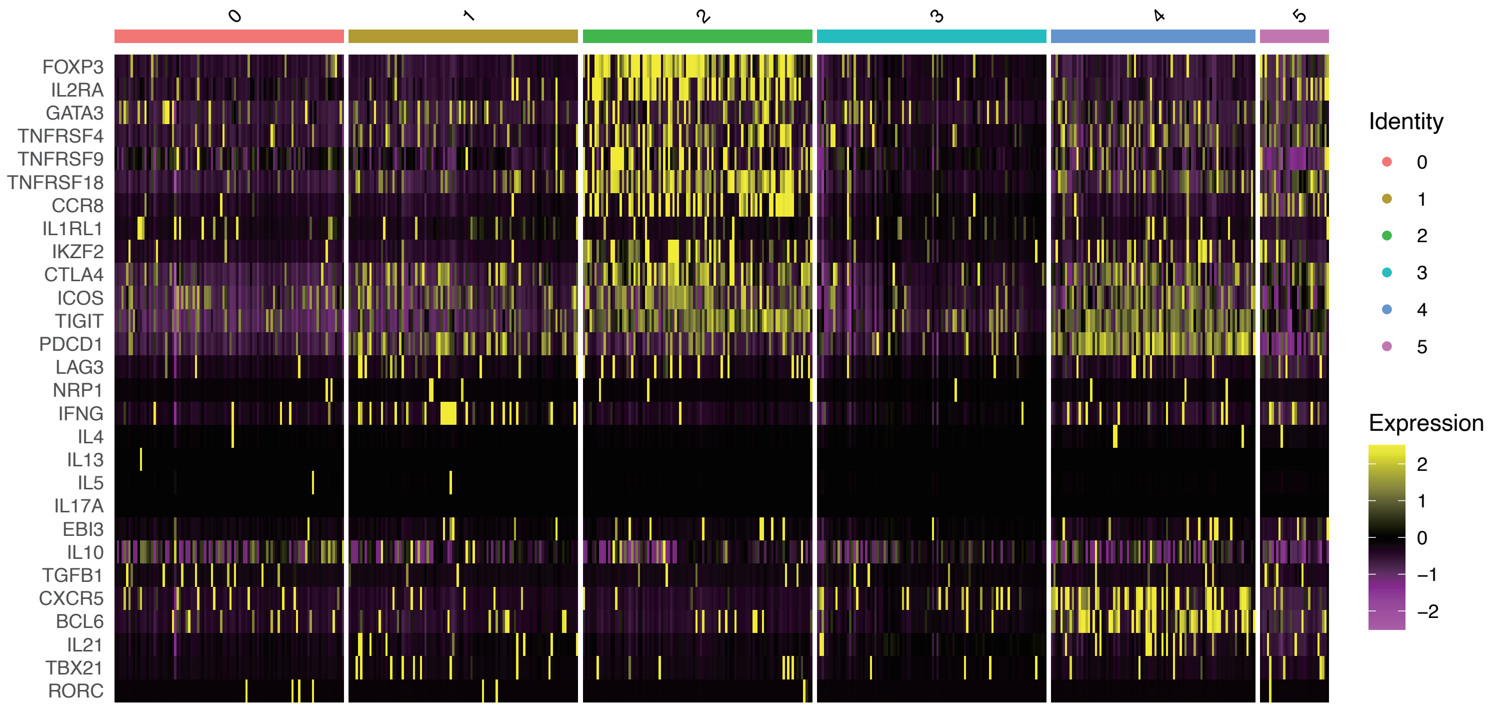

Supplemental Figure 12. Heatmap representation of the selected gene expression for the 6 clusters of CD4⁺ T cells retrieved from human melanoma dataset GSE115978. The cluster C2 exhibits featured expression of FOXP3, IL2RA, GATA3, TNFRSF4, TNFRSF9, TNFRSF18, CCR8, IKZF2, CTLA4, ICOS and TIGIT.

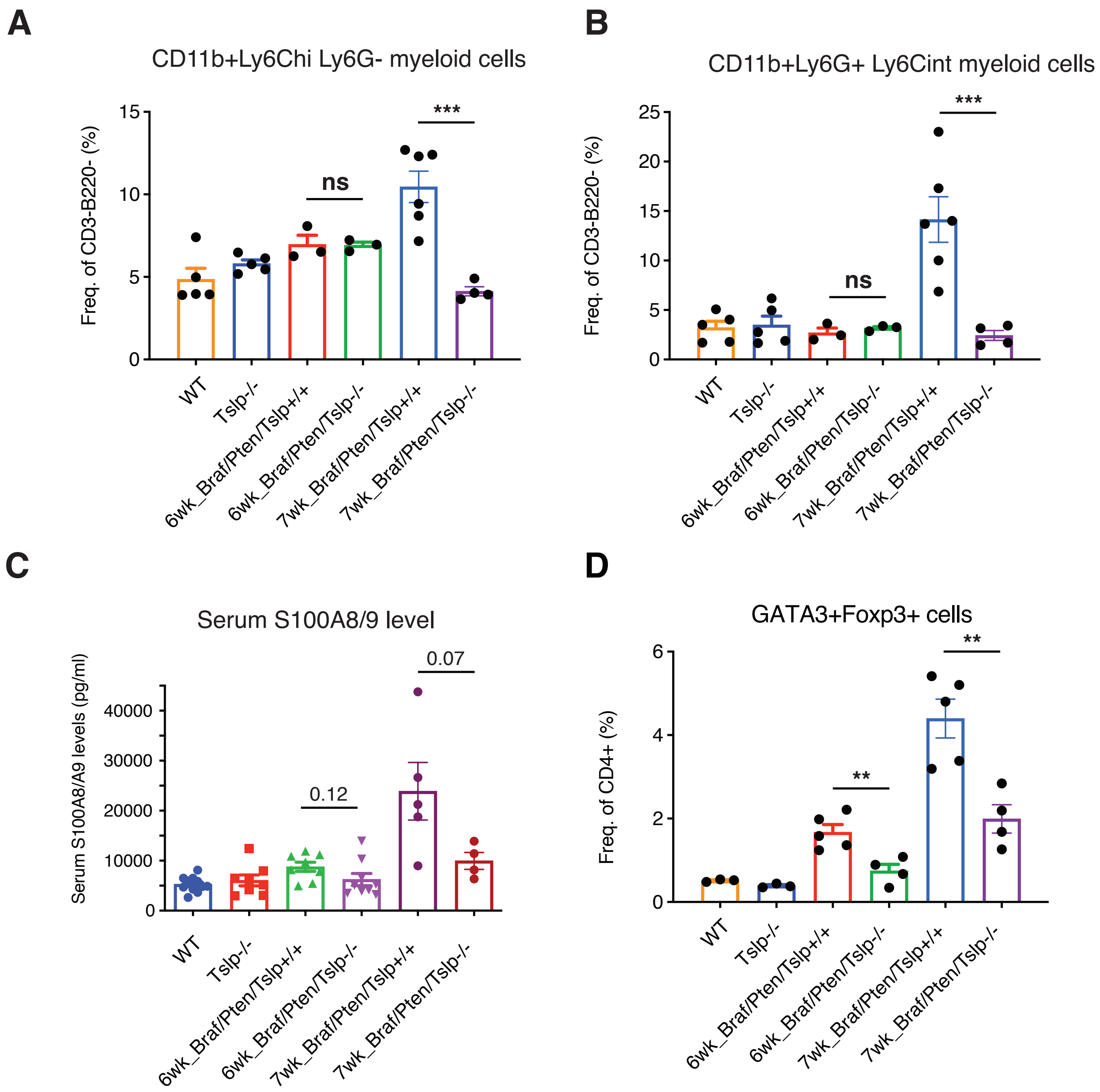

Supplemental Figure 13. (**A-B**) Comparison of the accumulation of CD11b^{+}Ly6C^{hi}Ly6G^{-} (M-MDSC) and CD11b^{+}Ly6G^{+}Ly6C^{int} (PMN-MDSC) cells in inguinal lymph nodes (ILNs) draining Braf/Pten/*Tslp*$^{+/+}$ or Braf/Pten/*Tslp*$^{-/-}$ dorsal tumors. Results showed that a significant difference for these cells between Braf/Pten/*Tslp*$^{+/+}$ and Braf/Pten/*Tslp*$^{-/-}$ mice was observed at 7 wks but not at 6 wks after 4-HT treatment. (**C**) Serum S100A8/A9 levels measured by ELISA (Mouse S100A8/S100A9 Heterodimer DuoSet kit; R&D; Cat. DY8596). (**D**) Comparison of GATA3^{+}Foxp3^{+} cell frequencies in ILNs, showing that the difference between Braf/Pten/*Tslp*$^{+/+}$ and Braf/Pten/*Tslp*$^{-/-}$ was already observed at 6 wks and continued to be seen at 7 wks after 4-HT treatment. Values are mean ± SEM. Students' t-test. *, p<0.05; **, p<0.01. ***, p<0.001. ns, non-significant.

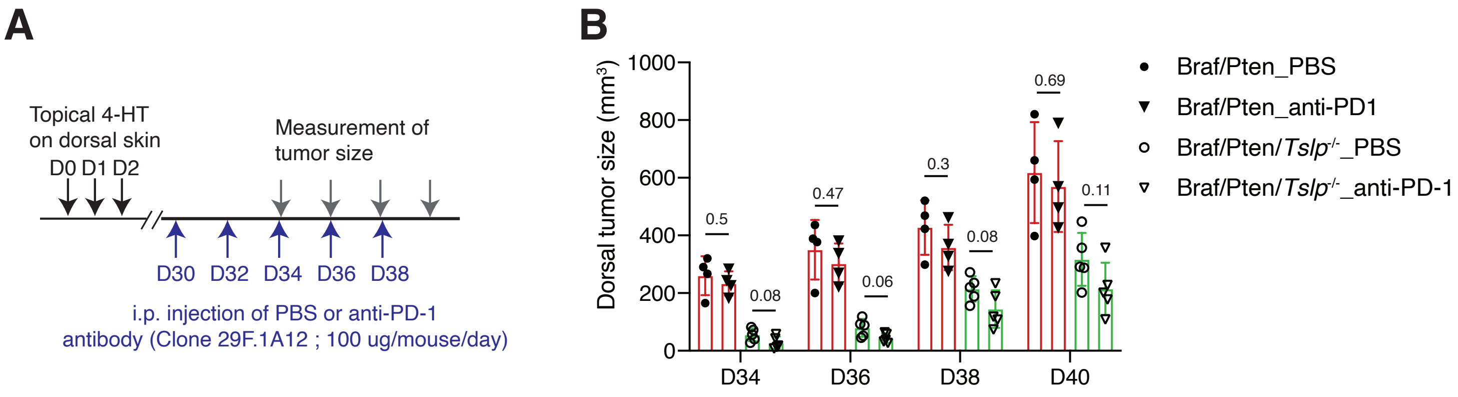

Supplemental Figure 14. Anti-PD-1 blockade in Braf/Pten and Braf/Pten/*Tslp*$^{-/-}$ mice. (**A**) Experimental protocol. Dorsal skin of Braf/Pten or Braf/Pten/Tslp-/- mice were topical treated with 4-hydroxytamoxifen (4-HT) for 3 days (D0-D2). Starting from D30, mice were i.p. injected with PBS or with anti-PD-1 antibody (Clone 29F.1A12; BioXCell; 100 µg/mouse/injection. Ref: Garris et al 2018 Immunity; PMID: 30552023) every other day. Dorsal tumor size was measured at D34, D36, D38 and D40. (**B**) Compasison of tumor sizes. Data are shown as each value (symbols) and their SEM (bars). Student's t-test. n=4 for Braf/Pten groups; n=5 for Braf/Pten/*Tslp*$^{-/-}$ groups. Data are representative of 2 independent experiments with similar resuts.

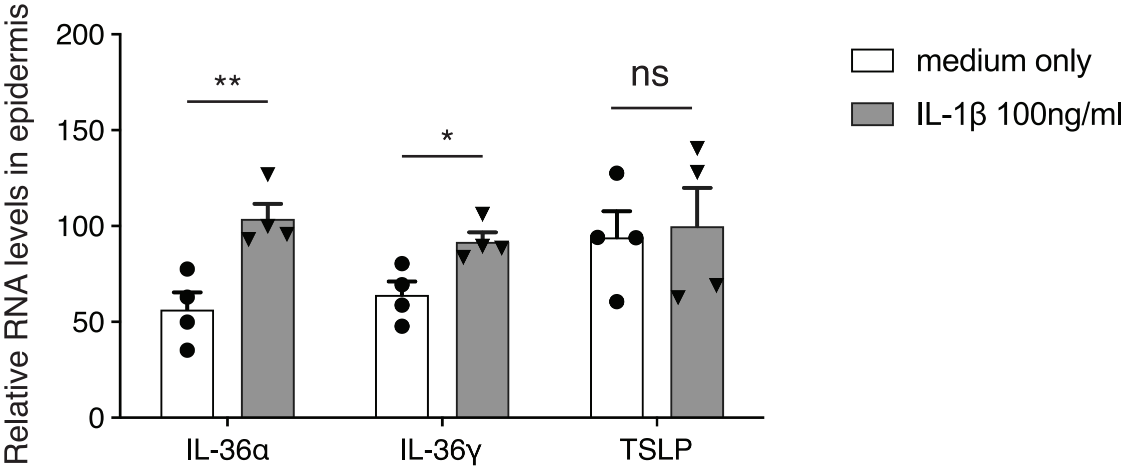

Supplemental Figure 15. IL-1β does not induce TSLP expression by *ex-vivo* cultured epidermis. RT-qPCR analyses of mouse epidermis ex-vivo cultured with IL-1β for 24 hrs, showing that TSLP RNA level is not induced, in contrast to increased levels of IL-36α and IL-36γ as positive controls for IL-1β induction. Values are mean ± SEM. Student's t-test. *, $p<0.05$; **, $p<0.01$; ns, non-significant.
